# Ultrafast Charge-Carrier Dynamics and Photocatalytic Mechanisms in Lanthanum-Doped Cerium Oxide Nanoparticles

**DOI:** 10.1021/acs.jpcc.6c05182

**Published:** 2026-09-30

**Authors:** Bach Pham, Claire A. Jones, Luke Kline, Meera Madhu, Bern Kohler

**Affiliations:** Department of Chemistry and Biochemistry, 2647 The Ohio State University, 100 West 18th Avenue, Columbus, Ohio 43210, United States

## Abstract

Cerium oxide nanoparticles (nanoceria) doped with trivalent lanthanum (La) ions and synthesized by a coprecipitation process at ambient temperature were studied by steady-state and femtosecond transient absorption spectroscopy to understand the photocatalytic mechanisms of this wide band gap photocatalyst in alkaline aqueous solution. UV irradiation reduces a significant fraction of cerium ions near the surface to Ce^3+^ centers that persist for hours in air. La doping increases the percentage of cerium ions that can be photoreduced, while also increasing the rate of photocatalytic degradation of an added dye molecule. Adding methanol eliminates dye photodegradation, showing that photogenerated hydroxyl radicals are the active species. Unexpectedly, the transient spectral signatures and dynamics of photogenerated electrons and holes are nearly unaffected by La doping. Fewer electron small polarons are formed in the La-doped nanoceria, but the dynamics of electron–hole recombination are unchanged during the 0–3 ns window probed. Furthermore, electrons and holes recombine according to a power law with an exponent that is inversely proportional to the Urbach energy determined from the steady-state absorption spectrum. These results are explained by a model in which a crystalline core of cerium oxide that lacks oxygen vacancies is surrounded by a defective shell that likely consists of an amorphous hydrous oxide phase containing Ce and La ions. The recombination of carriers trapped in this shell is unchanged on subnanosecond time scales, and La doping is proposed to enhance photocatalytic performance by enabling more holes to escape into the solvent via slow surface reactions.

## Graphical abstract

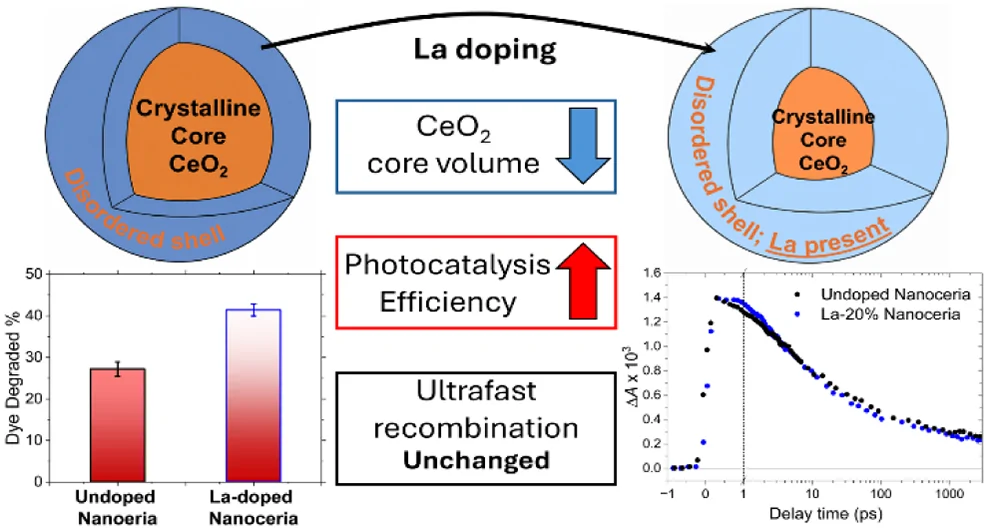

## 1 Introduction

Cerium­(IV) oxide (CeO_2_) nanoparticles (nanoceria) are a widely used catalyst in high-temperature applications such as solid-oxide fuel cells^1^ and catalytic converters^2^ due to excellent oxygen storage capacity and oxide ion conductivity.^3^ Nanoceria has also garnered attention for ambient temperature applications as a photocatalyst^3–5^ and as an enzyme-mimicking agent that can modulate the levels of reactive oxygen species in living organisms.^6–8^ For both low- and high-temperature applications, oxygen vacancies in nanoceria have been discussed as an important factor determining efficiency because this type of defect directly influences the oxygen storage capacity, conductivity, and surface reactivity of the material.^3,9,10^

Aliovalent rare-earth doping is a promising strategy for improving the photocatalytic ability of nanoceria to degrade organic dyes in aqueous solution, as shown by Liyanage et al. for Y-doped nanoceria.^11^ Later studies have explored doping with additional trivalent rare-earth dopants.^12–19^ Almost all of these works claim that the photocatalytic improvement originates from decelerated charge carrier recombination caused by oxygen vacancies induced by doping. Doping CeO_2_ with trivalent ions such as lanthanum (La^3+^) can introduce one oxygen vacancy for every two La^3+^ ions to maintain charge balance. Doping trivalent rare earth metal ions with La^3+^,^20,21^ gadolinium (Gd^3+^),^21,22^ and europium (Eu^3+^)^21,23^ is most often discussed as yielding solid solutions that retain the fluorite crystal structure of ceria. However, while oxygen vacancies are well documented in bulk CeO_2_,^24^ their existence in nanoceria synthesized by low-temperature precipitation methods and dispersed in water is uncertain. Even if surface oxygen vacancies can be formed, they are readily occupied by water or hydroxyl species in the aqueous dispersions used in photocatalysis experiments,^25,26^ limiting their availability as active defect sites. These observations raise questions about the effects of rare-earth doping on photocatalytic performance.

Here, we investigate a La-doped nanoceria photocatalyst prepared in a low-temperature synthesis through direct observations of electron–hole recombination on ultrafast time scales, augmented by steady-state measurements. Cerium oxide is an excellent model material for investigating polaron dynamics in metal oxide nanoparticles,^27–29^ and ultrafast transient absorption (TA) spectroscopy has proven particularly useful for investigating the charge carrier dynamics of few-nm ceria nanoparticles (nanoceria) dispersed in solvents due to the spectrally well-separated transient signals of electrons and holes^29^ but has not yet been applied to doped nanoceria. Time-domain observations are critically important for understanding the undesirable recombination of carriers and how these events are mediated by defects. In our opinion, paradigms about defect-mediated carrier recombination developed through study of materials with delocalized carriers are often applied inappropriately to polaronic materials, like nanoceria, in which charge carriers are highly localized. Our study aims to provide a fundamental connection between the charge carrier dynamics of nanoceria and its photocatalytic activity and to explore how recombination in polaronic nanoceria is affected by aliovalent rare-earth ion doping.

Using steady-state illumination from a Hg lamp, we study how La doping affects the accumulation of Ce^3+^ centers and the photocatalytic degradation of the dye Rhodamine B (RhB). With increased La-doping, a higher percentage of Ce^4+^ ions can be converted to Ce^3+^ by UV irradiation due to an expanded and defect-rich shell at the surface of the nanoparticles. Furthermore, we demonstrate that La-doped nanoceria prepared entirely at ambient temperature without high-temperature annealing degrades 25–40% more RhB molecules than undoped nanoceria under the same excitation conditions. We establish that hydroxyl radicals (•OH) formed by photogenerated holes are the main reactive species behind dye photodegradation. Although La doping increases the yield of redox-active •OH radicals, the rate of sub-ns recombination in nanoceria is essentially unchanged.

## 2 Experimental Section

All nanoceria samples were synthesized by coprecipitating Ce­(NO_3_)_3_·6H_2_O and La­(NO_3_)_3_·6H_2_O to achieve a La-to-Ce mole ratio of 0, 0.1, or 0.2. The resulting samples, which have poly­(acrylic acid) (PAA) capping ligands, are hereafter called undoped nanoceria, La-10% nanoceria, and La-20% nanoceria, respectively. The entire synthesis was carried out at ambient temperature without thermal annealing to minimize sintering and drying-induced aggregation. Steady-state and time-resolved experiments were performed by diluting a highly concentrated stock dispersion either with additional water or a mixture of 80% water and 20% methanol (v/v). Dispersions used in all measurements were adjusted to pH 10 with concentrated NH_4_OH (aq). Complete experimental details on synthesis and characterization are provided in SI Section 1.

## 3 Results

### 3.1 Characterization of La-Doped Nanoceria

TEM images (Figure S1) reveal spherical nanoparticles that are approximately 4 nm in diameter (Table S1) for all three samples (undoped, La-10%, and La-20%). The very small particle sizes minimize light scattering and enable transmission-mode TA measurements to be carried out with a high signal-to-noise ratio. All samples have similar DLS size profiles (Figure S2), indicating similar particle sizes in agreement with the TEM results on the dried powders. The hydrodynamic diameter of ∼10 nm determined by DLS is greater than the TEM diameter (Table S1) due to the PAA capping ligands.^30^ A similar DLS diameter of 12 nm was reported for undoped, PAA-capped nanoceria prepared using the same synthesis.^31^

XRF spectra (Figure S3) of the precipitated nanoceria samples confirm the presence of La in proportions that match the La:Ce mole ratio used in the synthesis (Table S2). XRD patterns (Figure S4, Table S3) show only peaks expected from the cubic fluorite structure of cerium oxide. The lattice parameters, crystallite sizes, and lattice strain estimated from the XRD patterns are listed in Table S4. Further details about the XRD analysis are in SI Section 3. Both raw and normalized Raman spectra (Figure S5) of all nanoceria samples show an asymmetric peak near 450 cm^–1^ and one or more weaker bands between 500–650 cm^–1^ that increase in amplitude relative to the 450 cm^–1^ band with increased La doping. The UV–vis spectra of the three nanoceria samples normalized to have a maximum amplitude of unity are indistinguishable within experimental uncertainty (Figure S6).

### 3.2 Photoreduction and Reactions of Undoped and La-Doped Nanoceria with H_2_O_2_

Absorption spectra of argon-sparged nanoceria dispersions with added methanol exhibit strong bleaching due to photoreduction in response to UV irradiation from a Hg lamp (Figure 1). The absorption decrease saturates with irradiation time, and the maximum photoreduction extent is defined as one minus the ratio of the lowest absorbance at 290 nm (*A*_290_) measured after irradiation to the value before irradiation. The maximum photoreduction extent increases from 44 ± 4% in undoped ceria to 53 ± 3% in La-10% nanoceria, and 65 ± 4% in La-20% nanoceria after 60 min of irradiation. A blue shift of the spectrum accompanies the photobleaching (Figure S7). After UV irradiation is stopped, *A*_290_ gradually grows back over hours with faster kinetics seen for samples exposed to air (Figure S8).

**Figure 1:**
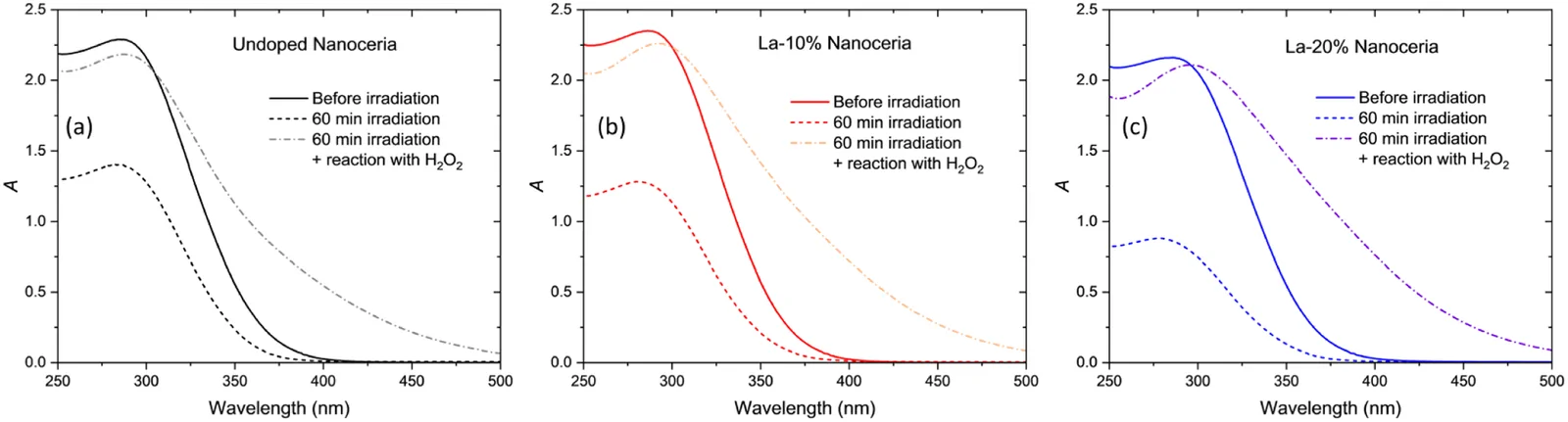
UV–vis absorption spectra of (a) undoped nanoceria, (b) La-10% nanoceria, and (c) La-20% nanoceria dispersed in water at pH 10 before and after irradiation with UV light (λ = 254 nm) for 60 min, and 30 min after adding H_2_O_2_ to the irradiated samples. To maximize photoreduction, methanol was added as a hole scavenger, and all samples were deaerated by argon sparging to remove dissolved oxygen.

To confirm that photobleaching in nanoceria is due to reduction of Ce^4+^ ions, aqueous H_2_O_2_ solution (30% w/w, Sigma-Aldrich) was added after irradiation to yield an initial H_2_O_2_ concentration of 1 mM. The mixture was allowed to react for 30 min until the absorption spectra (dot-dashed curves in Figure 1) stopped increasing. Reaction with H_2_O_2_ causes two changes to the absorption spectra. First, absorption at UV wavelengths less than 400 nm increases due to above band gap absorption as Ce^3+^ sites are oxidized to Ce^4+^. Second, a broad tail in the absorption spectrum extending to beyond 500 nm is seen, which is assigned to ligand-to-metal charge transfer from surface (hydro)­peroxo ligands.^32^ The absorption spectra recorded 30 min after adding H_2_O_2_ to the photoreduced samples have nearly the same maximum absorbance as before UV irradiation (Figure 1). Furthermore, adding H_2_O_2_ to samples that were not UV irradiated increased the maximum absorbance of each sample by no more than 2% (Figure S9), indicating that the fraction of cerium atoms in the +3 oxidation state is very low prior to UV irradiation. The highly oxidizing conditions of our synthesis at pH 10 are thought to leave the particles nearly fully oxidized.

### 3.3 TA Spectra of Undoped and La-Doped Nanoceria

TA spectra of undoped, La-10%, and La-20% nanoceria samples excited by 100 fs, 265 nm pump pulses recorded at various delay times are compared in Figure 2. The TA spectra exhibit the signature features of colloidal nanoceria with a narrow, more intense UV band accompanied by a broader, less intense visible band.^29^ The transient signal amplitudes can be meaningfully compared because the sample absorbance and pump fluence were adjusted (Table S5) to produce equal excitation densities in each sample. The initial amplitude of the UV band decreases significantly with increased La doping (Figure 2). On the other hand, the amplitudes of the visible band in the as-measured TA spectra are approximately equal when compared at the same delay time for all samples.

**Figure 2:**
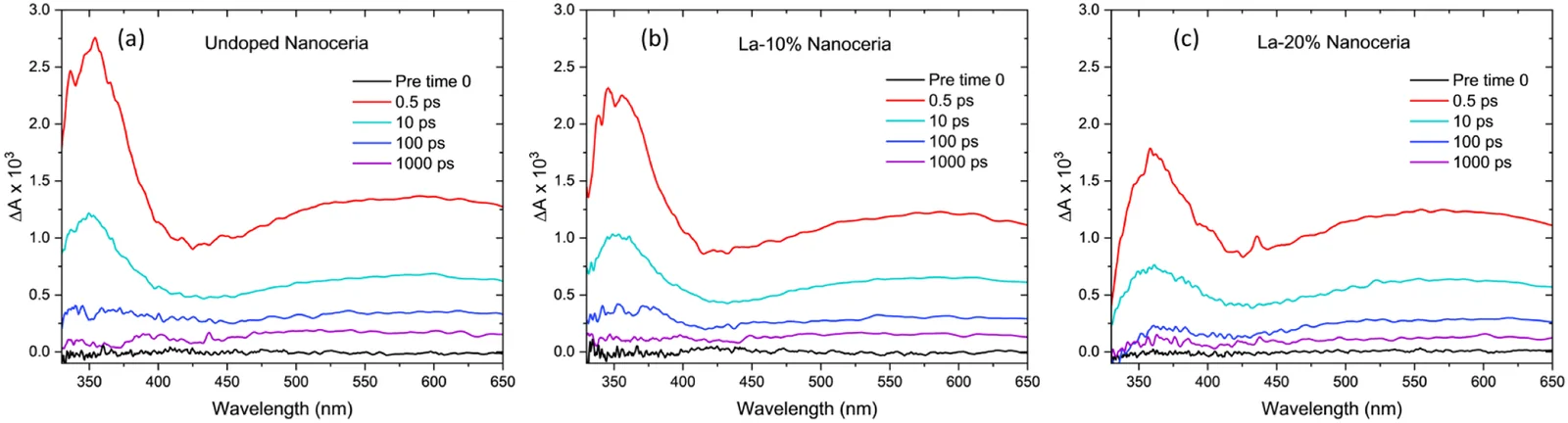
TA spectra recorded with excitation at 265 nm of (a) undoped nanoceria, (b) La-10% nanoceria, and (c) La-20% nanoceria at the indicated pump–probe delay times. All samples are dispersed in pH 10.0 water.

The trends in the TA spectra are confirmed in the TA kinetic traces, showing the measured absorbance change, Δ*A*, as a function of time, *t* (Figures 3 and 4). The UV band observed in all three samples has decayed to the baseline 200 ps after photoexcitation (Figure 3a). The decay kinetics of the UV band were fit to a stretched exponential function (eq 1),

1 $$\Delta A=A_{0}e^{-{\left(\frac{t}{\tau }\right)}^{\beta }}$$

where *A*_0_ is a dimensionless amplitude, *τ* is the characteristic decay time, and *β* is the stretching parameter. All fitting parameters are listed in Table S6.

**Figure 3:**
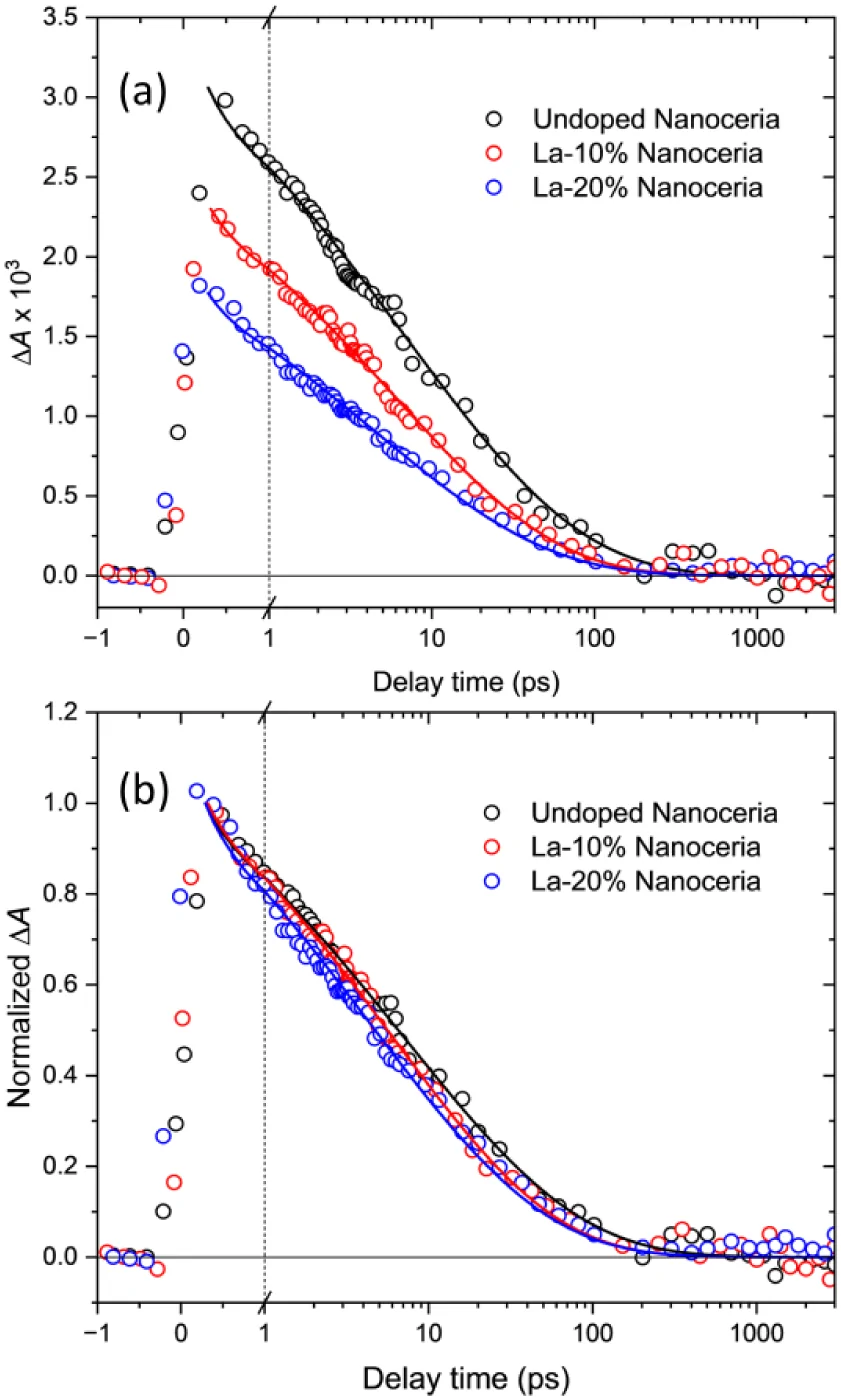
(a) As-measured and (b) normalized kinetic traces of the UV band of nanoceria samples dispersed in water at pH 10. The pump wavelength is 265 nm. The UV band traces are averaged over probe wavelengths from 350 to 360 nm and fit to a stretched exponential function (eq 1). In panel b, each kinetic trace and its best-fit curve were multiplied by a common scaling factor that makes the value of the fit curve equal unity at a delay time of ∼300 fs.

**Figure 4:**
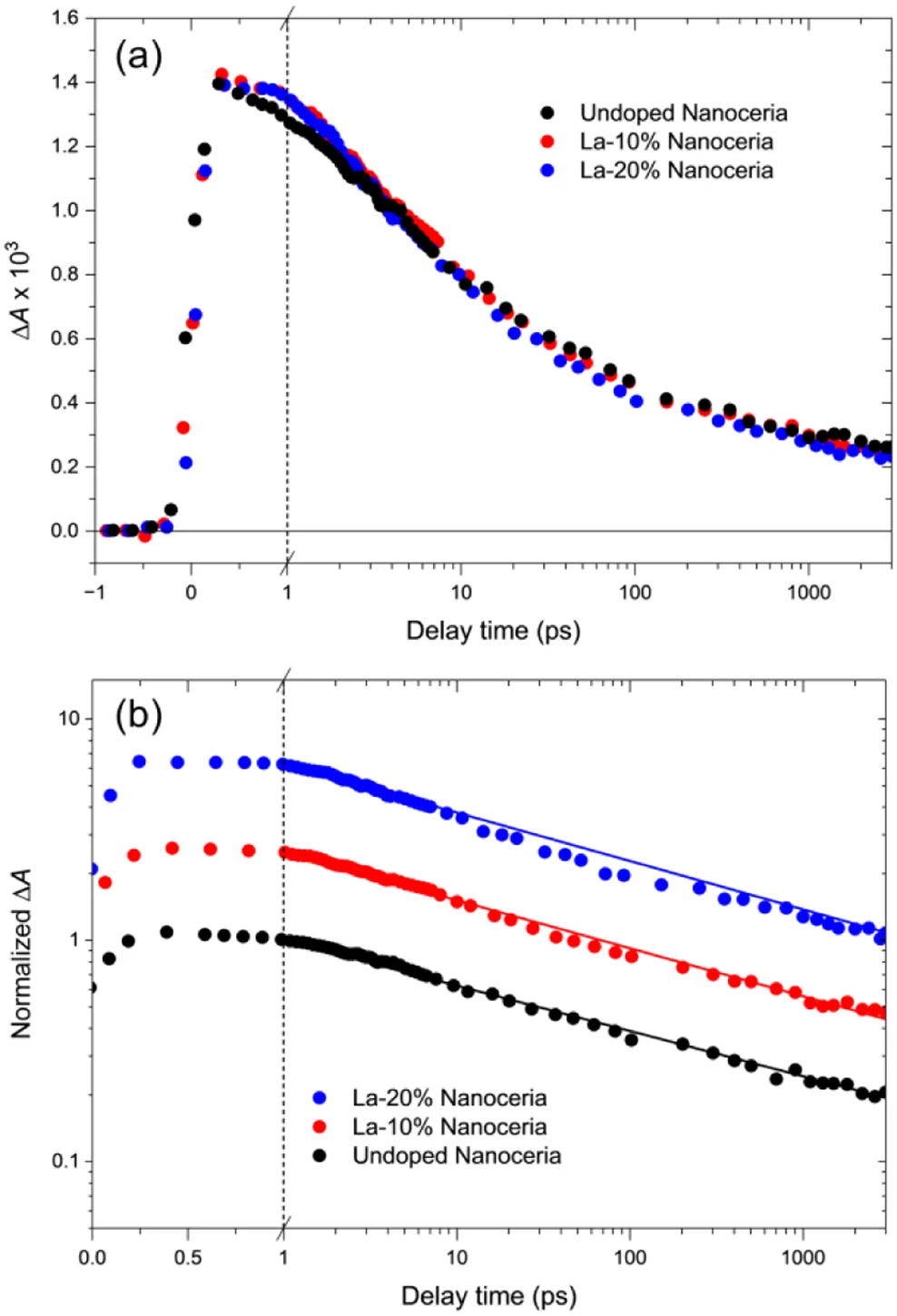
(a) Kinetic traces of the visible band assigned to surface-trapped holes. The three nanoceria samples are dispersed in pH 10 water, and the pump wavelength is 265 nm. Each trace is averaged over probe wavelengths from 500 to 600 nm. (b) Hole kinetics (markers) on a log–log plot showing power-law decays with approximately equal exponents at times >2 ps. The solid lines graph the power law function *t* ^–α^, where *α* is the exponent in eq 2. Each experimental trace was normalized to have unit amplitude at *t* = 1 ps, and the experimental and model kinetic traces for La-10% nanoceria and La-20% nanoceria were multiplied by 2.5 and 2.5^2^, respectively, for visual clarity. In all graphs, the time axis is linear at times <1 ps and logarithmic at later times.

The visible band kinetic traces agree well within experimental uncertainty (Figure 4a) and exhibit power-law decays only a few ps after *t* = 0 as seen from the linear appearance of the signals on a log–log plot (Figure 4b). The solid lines in Figure 4b are not least-squares fits to the markers but instead show the function,

2 $$N\left(t\right)\propto t^{-\frac{k_{B}T}{E_{U}}}$$

which represents the number of photogenerated holes, *N*(*t*), remaining at time, *t*. In eq 2, *k*_B_ *T* is equal to 0.0252 eV at the temperature, *T*, of 293 K at which the experiments were carried out, and *E*_U_ is the Urbach energy characterizing the energy distribution of traps. In Figure 4b, each data set was first scaled to have a value of 1 at *t* = 1 ps to equal the right-hand side of eq 2 at the same delay time. Subsequently, the markers and power-law curves for the two La-doped samples were scaled for visual clarity. The solid lines in Figure 4b are shown separately in Figure S10. The Urbach energy was determined from fits to the UV–vis spectra of the nanoceria samples shown in Figure S11 and described in SI Section 7. The implications of this model for electron–hole recombination in nanoceria are discussed in Section 4.3.

### 3.4 RhB Degradation Kinetics

The absorption spectrum of the nanoceria and RhB mixture agrees with the summed spectra of nanoceria and RhB recorded separately (Figure S12). The performance of the nanoceria photocatalysts was assessed by irradiating an aqueous, pH 10 solution containing RhB and the dispersed nanoceria samples with UV radiation from a Hg lamp. The concentration of the dye was measured periodically by pausing the irradiation and measuring the solution absorbance at 554 nm (Figure 5), as absorption at this wavelength under steady-state conditions originates solely from the dye. In addition, no RhB degradation is observed when the dye is present with oxidized or reduced nanoceria in the dark (Figure S13).

**Figure 5:**
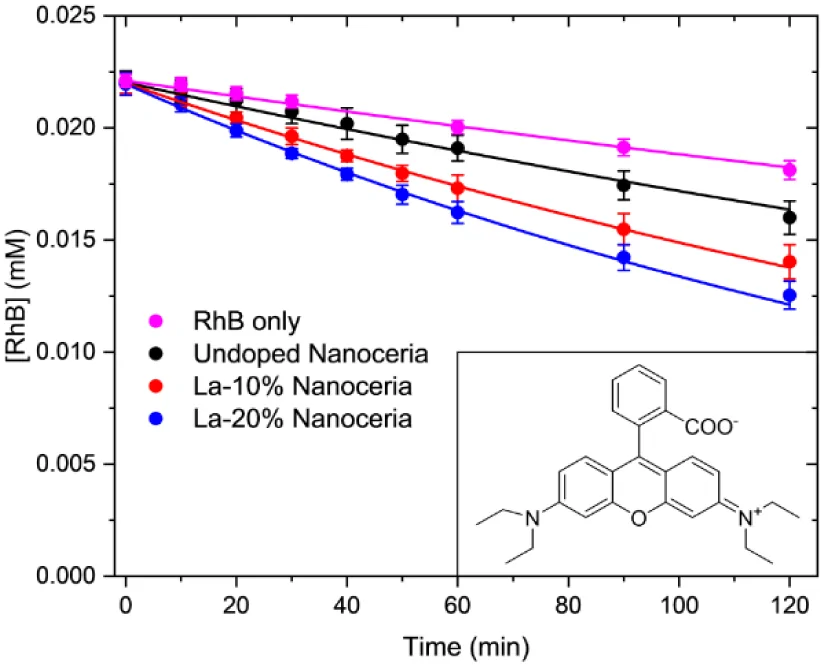
Kinetics of dye degradation induced by Hg lamp irradiation (λ = 254 nm). The RhB concentration is calculated from the dye absorbance at 554 nm using a molar absorption coefficient of 79,600 M^–1^ cm^–1^. The degradation kinetics of RhB in the absence of the photocatalyst are shown for reference (magenta markers). Error bars are 2σ estimates from 5 trials. Solid curves are fits to a first-order decay model. (Inset) Chemical structure of RhB in the zwitterionic form present at pH 10.

The absorption spectra after 0 and 120 min of UV irradiation are compared in Figure S14. Absorption at visible wavelengths in the nanoceria and dye mixture is identical to absorption by the dye alone. The bleaching of the visible band, which is greatest in the La-20% nanoceria sample, thus indicates dye degradation. The percentage degraded after 120 min irradiation increases from 27 ± 2% in undoped ceria to 33 ± 2% in La-10% nanoceria and 39 ± 2% in La-20% nanoceria. Here, the stated uncertainties are 2σ estimates from 5 trials using nanoceria samples that were freshly synthesized each time.

Irradiating an aqueous, pH 10 solution of 0.022 mM RhB in the absence of the photocatalyst in the Hg lamp photoreactor causes the absorbance to decrease by approximately 20% after 2 h of irradiation (Figure 5, pink markers). The self-degradation of the dye likely occurs through singlet oxygen generation by its lowest triplet excited state as shown for other rhodamine derivatives.^33^ Because nanoceria in the dye mixture absorbs ∼85% of the 254 nm radiation from the Hg lamp, a strong inner filter effect exists that reduces the rate of dye self-degradation when the photocatalyst is present.

The spectra in Figure S14 reveal further that absorbance changes at UV wavelengths <300 nm are greater than the initial absorbance of the dye in this spectral region, indicating that the Hg lamp irradiation bleaches the nanoceria in addition to degrading the dye. This is confirmed by the graphs in Figure S15, showing the absorbance due to the dye and nanoceria as a function of irradiation time, assuming that the absorbance by the mixture is additive in the two components at all times.

Adding methanol, an •OH radical scavenger, significantly slows down the dye degradation kinetics of all nanoceria samples (Figure 6). The percentage of dye degraded after 2 h decreased by ∼70% when methanol is present (Figure 7). Neither the removal of dissolved oxygen (DO) by argon sparging (Figure S16) nor the addition of an electron scavenger (AgNO_3_, Figure S17) measurably affects the dye degradation kinetics.

**Figure 6:**
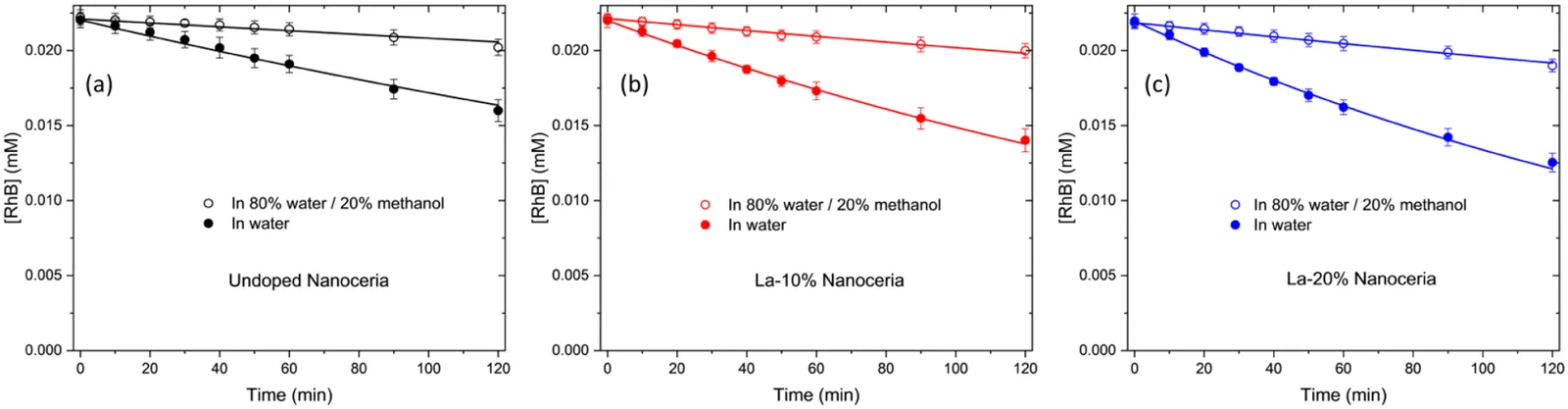
Dye degradation kinetics of (a) undoped nanoceria, (b) La-10% nanoceria, and (c) La-20% nanoceria samples under Hg lamp irradiation (λ = 254 nm) in neat water (filled circles) and in an 80% water/20% methanol mixture (empty circles). Solid curves are fits to a first-order decay model. Error bars are 2σ estimates from 5 trials.

**Figure 7:**
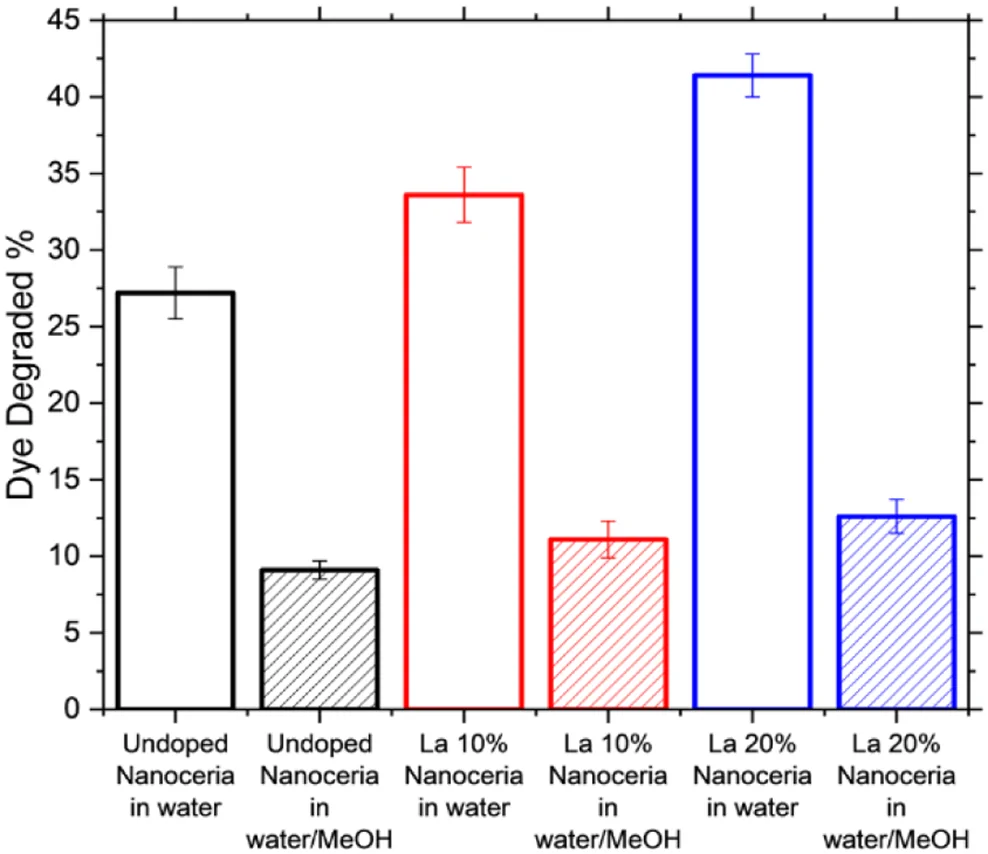
Percentage of dye degraded after 120 min of Hg lamp irradiation in pH 10 aqueous solution (clear bars) compared to an alkaline 80% water, 20% methanol mixture (hatched bars; percentages are volume fractions). Error bars are 2σ estimates from 5 trials.

### 3.5 Quantification of •OH Formed by Irradiated Nanoceria

A fluorescence assay was used to detect •OH radicals produced by photoexcitation of nanoceria. These radicals could be freely moving through the aqueous solution or they could be surface-bound radicals that escape recombination long enough to react with a probe molecule. In these experiments, aqueous nanoceria dispersions containing 0.1 mM coumarin were irradiated with the Hg lamp. Coumarin is nonfluorescent but can form the highly fluorescent product 7-hydroxycoumarin (7HC) by reaction with •OH. Of the several possible products, only 7HC is fluorescent.^34^ The absorption maximum of 7HC is at 370 nm,^35^ where nanoceria absorbs weakly, allowing its emission to be recorded without inner filter effects. After irradiation, emission spectra were recorded with 370 nm excitation (Figure 8a). Using an authentic sample of 7HC, a calibration curve relating the emission intensity to concentration (Figure S18) was used to estimate the concentration of 7HC formed (Figure 8b).

**Figure 8:**
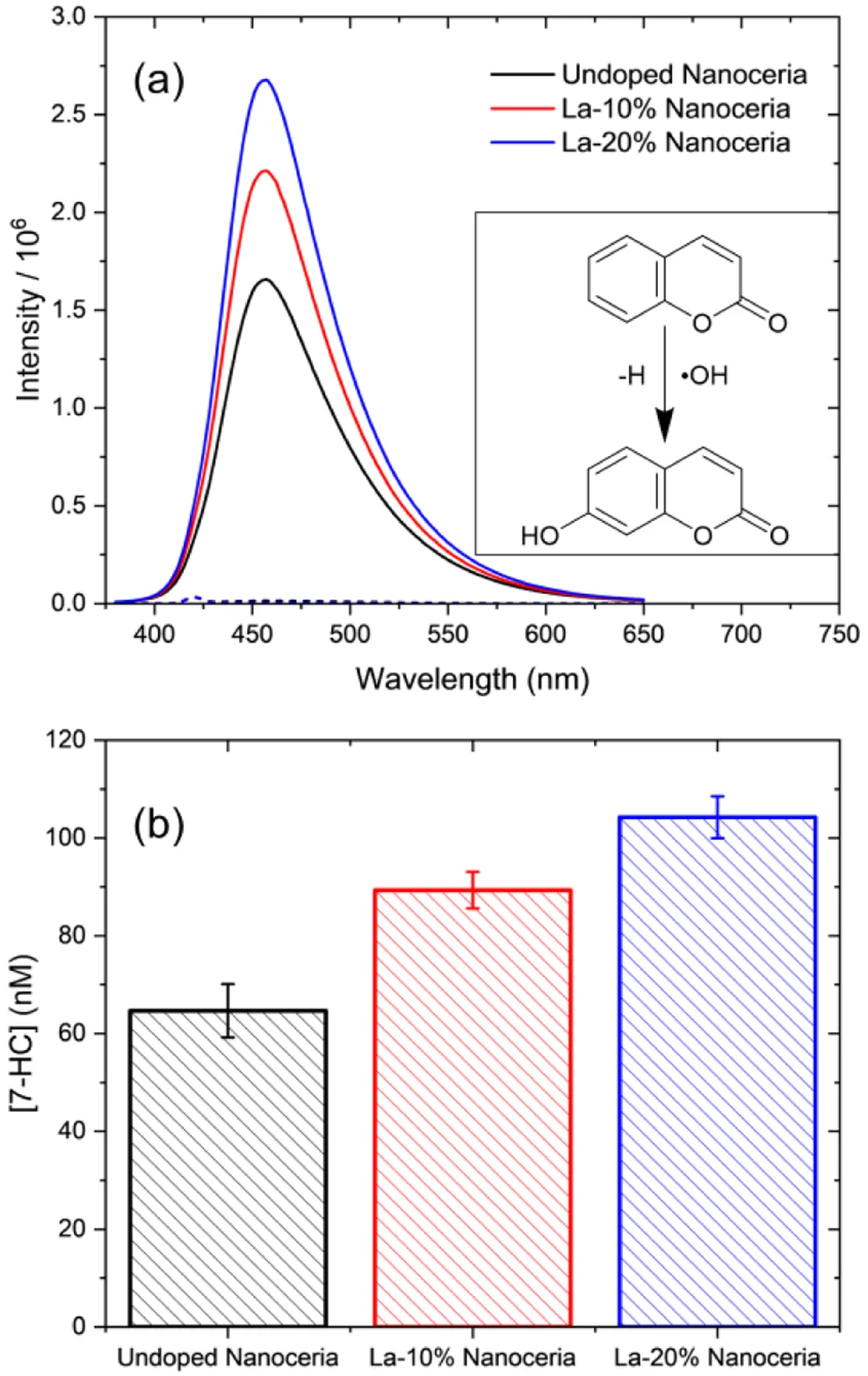
Detection of 7HC in UV-irradiated nanoceria dispersions containing 0.1 mM of coumarin. (a) Emission spectra of each nanoceria-coumarin dispersion excited at 370 nm before (dashed lines) and after (solid lines) 30 min of irradiation. (Inset) Chemical structure of coumarin (above) and the emissive product 7HC (below) formed by reaction with •OH. (b) Concentration of 7HC after 30 min of irradiation determined from the calibration curve in Figure S18. Error bars are 2σ estimates from 5 independent trials.

Adding methanol to the nanoceria and coumarin mixture results in much weaker and red-shifted emission (dashed lines in Figure S19a). This weak emission agrees well with the emission measured when a pH 10 solution of PAA is irradiated by itself in the photoreactor (Figure S19b). This shows that methanol quenches the photogenerated hydroxyl radicals before they can react with coumarin in the solution. The quantum yield of •OH formation (Table 1) calculated from the measured amount of 7HC and the absorbed photon flux determined through actinometry (Figure S20 and SI Section 10) increases by ∼70% from undoped nanoceria to La-20% nanoceria.

**Table 1:** Concentration of 7-Hydroxycoumarin, [7HC], Detected After 30 min of Hg Lamp Irradiation, Concentration of •OH, [•OH]_total_ After 30 min of Hg Lamp Irradiation, and the Quantum Yield of •OH, *Φ*_•OH_, Calculated as Discussed in SI Section 10

Samples	[7HC] (nM)	[•OH]_total_ ^a^ (nM)	*Φ*_•OH_
Undoped Nanoceria	64 ± 5	1000 ± 100	0.006%
La-10% Nanoceria	89 ± 4	1500 ± 100	0.009%
La-20% Nanoceria	104 ± 4	1700 ± 100	0.010%

a Calculated assuming that 6.1% of OH radicals yield 7HC.^34^

## 4 Discussion

### 4.1 Effects of La Doping on Nanoceria Structure

The XRF spectra (Figure S3) indicate that La ions have been successfully incorporated into the nanoceria in the desired La:Ce mole ratio (Table S2). The XRD patterns provide important clues about the structure of our nanoparticles and are analyzed in detail in SI Section 3. Crystallite sizes and lattice microstrain were extracted from Williamson-Hall plots (Figure S4b). The trend in sizes agrees with Scherrer equation estimates. While the average crystallite size decreases from 3.3 nm in undoped nanoceria to 2.7 nm in La-10% nanoceria and 2.4 nm in La-20% nanoceria, the lattice microstrain remains constant in all samples within experimental uncertainty (Table S4). In metal oxides, the lattice microstrain is expected to increase when dopants with mismatched radii are incorporated into the crystal lattice.^36,37^ The unchanging lattice microstrain regardless of La doping thus suggests that the crystalline core of the nanoceria is not disrupted by defects such as La^3+^ ions, Ce^3+^ ions, or oxygen vacancies.

The introduction of trivalent dopants leads to a lattice expansion in bulk cerium oxide if the dopant ionic radius is larger than the critical dopant radius of 1.027Å.^38^ La^3+^ with an ionic radius of 1.17 Å^39^ should thus expand the lattice, and it is known from Rietveld analysis that the ceria lattice expands to accommodate the larger La^3+^ ions.^40^ However, the lattice parameter obtained from the XRD peaks in our samples (Table S4) increases by only ∼0.15% for La-20% nanoceria. We explain the minimal lattice expansion seen in our samples by a core–shell model in which a core of crystalline CeO_2_ is surrounded by a disordered shell containing Ce^3+^/Ce^4+^ and La^3+^ ions. Due to its amorphous character, the shell is assumed to not contribute to the XRD signals. Ce atoms in the shell must be overwhelmingly in the +4 oxidation state in the as-synthesized nanoparticles based on the results of adding H_2_O_2_ (Figure 1). We argue in the next section that UV irradiation allows most or all of the Ce^4+^ ions in the shell to be reduced to Ce^3+^.

Studies of La-doped cerium oxide samples subject to high temperature (800 K–1300 K) conditions during^41^ or after^42^ the oxide formation phase show that the lattice parameter expands up to 2%, considerably more than the expansion of ∼0.15% in our samples, which were not heat treated. High-temperature annealing was reported to cause lattice expansion of 1.5% for 8–15 nm nanocrystals^42^ and 1.3% for 20–50 nm nanocrystals.^43^ High-temperature treatment allows La^3+^ ions that are likely constrained by synthesis to be initially near the particle surface to diffuse into the bulk together with charge-compensating oxygen vacancies. The oxygen vacancy migration barrier calculated by DFT+U^44,45^ and HSE06^45^ is 0.49 eV–0.53 eV, which means that a temperature increase from 293 to 1000 K increases the oxygen vacancy mobility by approximately 6 orders of magnitude. TiO_2_ has been shown to form more bulk oxygen vacancies when the synthesis is conducted at elevated temperature.^46^

We propose that La^3+^ ions occur only in the defective surface region in our La-doped nanoceria. Even in thermally annealed samples, La ions are enriched at the surface of small nanoparticles compared to the bulk, especially for low doping levels.^41,42,47–49^ A possible explanation for the surface segregation of La^3+^ in nanoceria is the ion’s higher basicity compared to Ce^4+^. Because the metal oxide phase is formed through the hydrolysis of the metal ions, the slower rate of hydrolysis by the more basic La^3+^ ions likely underlies surface segregation.^42^

Room-temperature synthesis also curtails the mobility of oxygen vacancies, essentially immobilizing them in the surface shell. In refs 41,42,47 , and^49^, the surface composition was determined using XPS, which has a reported probing depth of 1.5 nm.^41^ Bellière et al. reported that for Ce_1–*x*_La_*x*_O_2‑*x*/2_ samples with overall composition of *x* = 0.10 and 0.20, 22% and 39%, respectively, of metal ions at the surface are La.^41^ Reddy et al. showed that 42% of metal atoms at the surface of their CeO_2_ samples with an overall La content of 20% are La atoms.^42^ These studies examined La-doped nanoceria that was either synthesized hydrothermally at an elevated temperature or that underwent thermal annealing. Studies of the precise distribution of La^3+^ ions in samples like ours, which were synthesized entirely at ambient temperature, are desirable but exceed the scope of this study.

The precise nature of the phase in the shell region is uncertain, but it must contain Ce in addition to La based on the photoreduction experiments discussed in Section 4.2. If La^3+^ substitutes for Ce^4+^ in an amorphous ceria phase, oxygen vacancies could be present, but charge compensation could also be provided by intercalated protons.^50,51^

Raman spectra (Figure S5) provide additional information about the structure of La-doped nanoceria. The F_2g_ band seen at 450–455 cm^–1^ arises from the symmetric vibration of the Ce–O_8_ units found in the crystalline fluorite structure.^52,53^ This band is red-shifted compared to the 465 cm^–1^ band seen in bulk CeO_2_ crystals^54^ and broadened asymmetrically because of the small crystallite size, defects, and surface relaxation.^53^ The amplitude of the F_2g_ band in our La-doped nanoceria is lower than in our undoped nanoceria (Figure S5a), consistent with a crystalline core that shrinks with increased La doping (Figure 9). Wu et al.^55^ showed that smaller nanocrystals exhibit less intense F_2g_ peaks, supporting our interpretation.

**Figure 9:**
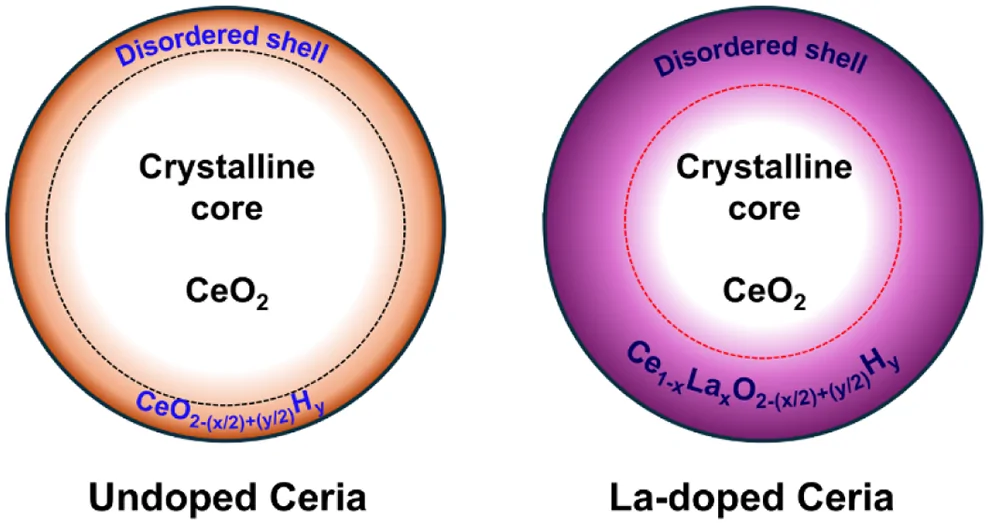
Schematic illustration of how La doping extends the thickness of a defective and possibly amorphous shell, which surrounds a crystalline CeO_2_ core that is free of metal ion dopants. The possible composition of the shell, which is likely to be extensively hydroxylated, is discussed further in SI Section 8.

La doping of CeO_2_ introduces a shoulder band in the Raman spectra of the solid powder between 500–650 cm^–1^ (Figure S5),^54–57^ which has been called the defect band^53^ or defect-induced mode.^55^ This mode is inactive in a perfect fluorite structure and only becomes Raman active when defects lower the symmetry.^53,58^ As more La is incorporated into the nanoceria, this defect band grows in intensity (Figure S5), consistent with an increased number of defects. However, the exact origin of the defect band is still debated. Some investigators assign this band to O vacancies and/or O vacancy-dopant ion complexes as reviewed in refs 58 and 59. However, Wu et al.^55^ suggest that Frenkel-type interstitial oxygen defects created when oxygen ions relocate from tetrahedral to octahedral sites are responsible. However, these proposals, which emphasize defects detected in bulk crystals at high temperature or UHV conditions, may not apply to few-nm nanoparticles in aqueous solution. In the latter conditions, a large fraction of metal ions reside on the surface, and vacancies can be readily filled by (possibly dissociated) water molecules. As discussed elsewhere,^29,51^ protons can be drawn into the surface region, providing charge compensation and giving rise to hydrous oxide phases. The uncertainties and apparent contradictions in the Raman assignments would benefit from a comprehensive Raman spectroscopy investigation of colloidal nanoceria, but this is beyond the scope of this study.

We conclude that growth of Raman scattering from 520–650 cm^–1^ indicates that a growing number of defects are formed by La doping, but the XRD results discussed above suggest that these defects are located overwhelmingly in the surface region and may be distinct from the O vacancies found in bulk or high-temperature ceria. TEM images were acquired in bright-field mode and sizing at this magnification is sensitive to the total particle diameter. The crystallite sizes obtained from the XRD analysis are consistently smaller than the TEM diameters, consistent with a crystalline core region that contracts with increased La doping. All evidence points to a crystalline CeO_2_ core that is surrounded by a disordered La-doped shell that is likely extensively hydroxylated (Figure 9). We discuss in the following sections how the core–shell model explains the photoreduction behavior and ultrafast spectroscopic observations and provides insights into the photocatalytic mechanism and performance.

### 4.2 Photoreduction of La-Doped Nanoceria

UV irradiation of undoped nanoceria produces electron–hole pairs, and the electrons self-trap to form electron small polarons (ESPs). Each ESP consists of a Ce^3+^ ion and a distorted shell of neighboring oxide ions.^27,29^ Holes trap to the surface in <1 ps, while ESPs in the crystalline interior of nanoceria with diameter of ∼4 nm move to the surface in <100 ps by hopping because this is where the deepest traps should exist.^29^ If the hole reacts with a water or alcohol molecule in the solvent phase, then a Ce^3+^ center can avoid recombination and persist for hours. The reduction of many Ce^4+^ ions in each nanoparticle to Ce^3+^ decreases the absorbance at UV wavelengths by 40% or more (Figure 1), and this decrease can be used to assess the Ce^3+^ content of nanoceria.^51,60^ Strong CT absorption from oxide ions to Ce^4+^ ions is responsible for nanoceria’s above band gap absorption at UV wavelengths, while Ce^3+^ ions contribute negligibly.^60^ The absorbance at UV wavelengths is thus proportional to the moles of Ce^4+^ ions in the dispersion.^51,60^

The maximum photoreduction extent of the samples increases with greater La doping (Figure 1). Because La doping decreases the volume of the crystalline core (Section 4.1), this indicates that Ce^3+^ ions are accumulating in the expanding shell region. Under conditions that maximize photoreduction, i.e., deoxygenated solutions with an added hole scavenger (methanol), most of the surface of undoped nanoceria can be reduced.^29,60^ Adding hydrogen peroxide shows that the samples have minimal Ce^3+^ content before UV irradiation (Figure S9). This suggests that the maximum photoreduction extent is determined by the fraction of all Ce atoms found in the shell region. The ratio of Ce ions in the surface shell to total Ce ions (see Table S7 and SI Section 8) is 47% for undoped nanoceria, 61% for La-10% nanoceria, and 68% for La-20% nanoceria. These values agree reasonably well with the maximum photoreduction extent of 44 ± 4%, 53 ± 3%, and 65 ± 4% for the corresponding samples. This agreement supports our conclusion that the increase in photoreduction extent is caused by a surface shell that increases in thickness with increased La doping and that is disordered enough to stabilize large numbers of Ce^3+^ ions through oxide ion protonation.

### 4.3 Effects of La Doping on Charger Carrier Dynamics

The La-doped nanoparticles exhibit the same two bands seen in the TA spectra of undoped nanoceria (Figure 2), namely, a broad band at visible wavelengths (“visible band”) and a narrower band centered near 350 nm (“UV band”).^29^ This indicates that La doping does not alter the spectral signatures and identities of the charge carriers. We assign the UV band to bulk ESPs resulting from the self-trapping of electrons in the crystalline bulk upon photoexcitation.^29^ ESPs in crystalline ceria form by electron self-trapping within 300 fs.^27,29^ As mentioned previously, the ESPs migrate to the defect-rich shell region by thermally activated hopping between Ce^4+^ sites. Once they are in the amorphous shell region, the deeply trapped ESPs or Ce^3+^ centers no longer contribute to the UV band, consistent with the steady-state photobleaching experiments. The UV band thus indicates that ESPs are present in the crystalline CeO_2_ core.

The visible TA band is assigned to holes that have localized in traps on the nanoceria surface.^29^ Unlike electrons, holes in nanoceria are unable to self-trap in the crystalline bulk at ambient temperature, but they trap to the surface of nanoceria in <300 fs.^29^ The visible band rises within the time resolution of our instrument in all samples. Surface hydroxy groups have been proposed to trap holes in TiO_2_, forming surface •OH radicals.^61,62^ Therefore, the visible signal is presumed to track the population of surface-adsorbed •OH with one such radical formed for every absorbed photon. Most surface holes will recombine with their geminate electrons or oxidize PAA ligands but a few can persist long enough to contribute to photocatalytic reactions.

#### 4.3.1 ESP Dynamics in La-Doped Nanoceria

The observed decrease in the initial UV band amplitude in Figure 2 indicates that fewer bulk ESPs are formed as La doping increases. In the most heavily doped La-20% nanoceria, the initial UV band amplitude measured by the fitting parameter *A*_0_ listed in Table S6 is 63% as large as in the undoped sample. This decrease is explained by a decrease in the crystalline core volume due to the expanding disordered shell (Figure 9). Because only ESPs in the crystalline core contribute to the UV band, the initial band amplitude is proportional to the fraction of cerium atoms that are in the core vs shell (Figure S21). The decreasing band amplitude thus indicates that the core decreases in size with increased La doping.

The behavior of the initial amplitude of the UV band mirrors that of the F_2g_ Raman peak intensity, which decreases in intensity as the core shrinks (Figure S5a). The F_2g_ Raman band is broad and asymmetric in as-deposited ceria thin films, which have amorphous microstructure.^63^ Weak F_2g_ scattering was also reported for an amorphous oxyhydroxide/peroxide phase formed when hydrogen peroxide reacts with nanoceria.^64^ A shrinking core of crystalline CeO_2_ and an amorphous shell of La-doped ceria is thus expected to decrease the intensity of the F_2g_ band as observed.

The normalized UV band kinetic traces appear similar on a log time axis (Figure 3b), but the average decay time ⟨*τ*⟩ of the UV band calculated from the best-fit parameters (Table S6) decreases modestly with increased doping. The characteristic decay time of the UV band in undoped nanoceria corresponds to the time needed for the ESP to be transported by hopping to deep traps in the disordered surface shell. This time is proportional to *R* ^2^, where *R* is the radius of the crystalline core of each sample.^29^

We propose that the faster decay of the UV band with increased La doping reflects the reduced time needed for the hopping ESP to transit the crystalline core and reach the defective shell region, where it no longer contributes to the UV band. Taking the radius *R* of the crystalline core of each sample as half the crystallite size determined by XRD analysis (Table S4), the average transit time obtained from the average decay time ⟨*τ*⟩ of the UV band (Table S6) varies approximately linearly with *R* ^2^ (Figure S22). The diffusion coefficient of the bulk ESP derived from the slope of the best-fit line is (0.7 ± 0.3) × 10^–4^ cm^2^ s^–1^, which is somewhat smaller than our previously reported value of (1.5 ± 0.2) × 10^–4^ cm^2^ s^–1^.^29^ The deviation likely arises from the smaller radius definition used here. In our previous work,^29^ *R* was obtained from the TEM size, which is larger than the XRD value due to the thickness of the defect-rich surface shell.

#### 4.3.2 Hole Dynamics and Surface Recombination

Unlike the UV band, the initial visible TA band amplitude is independent of the level of La doping (Figures 2 and 4a). The initial excitation may decohere into an ESP in the particle interior and a surface hole on the femtosecond time scale. Trapping of the latter may be due to coupling to surface hydroxyl groups that are similar in undoped and La-doped nanoceria.

La doping does not significantly affect the visible signal decay kinetics between 0–3 ns (Figure 4a). Trapping-detrapping models with an exponential distribution of trap states predict the carrier survival probability to decay asymptotically as a power law (i.e., as *t* ^–α^).^65,66^ The early onset of the power-law decay only a few ps after excitation of all samples (Figure 4b) is suggested to arise from the subpicosecond trapping of electrons and holes and their subsequent movement through an energetically disordered landscape.

The decay of the hole signal through electron–hole recombination between ∼2 ps and 3 ns is predicted nearly quantitatively by power law decays with $\alpha =\frac{k_{B}T}{E_{U}}$ (solid lines in Figure 4b), where *E*_U_ is extracted from the steady-state absorption spectrum of each sample (Figure S11). The excellent agreement indicates that recombination in nanoceria with and without La-doping is determined by the release of carriers from surface traps that are responsible for the tailing (Urbach tail) in the absorption spectrum. These traps are proposed to be more numerous in the La-doped shell, but to be energetically similar to ones in undoped nanoceria.

The unchanging decay of the visible band assigned to surface holes indicates that electron–hole recombination occurs at the same rate over sub-ns time scales, and this observation supports the conclusion that the nanoparticle core is free of La^3+^ ions. Janotti and coworkers have calculated that the activation energy for hopping by an ESP bound to an O vacancy in crystalline CeO_2_ is ≥0.26 eV.^45^ If the activation energy in La-doped ceria is similar, any oxygen vacancies present in the core to compensate the charge of La^3+^ ions would cause the ESPs to migrate much more slowly to the nanoparticle surface, but this is not observed. These considerations support the model in Figure 9 in which the core of the La-doped nanoparticles is crystalline CeO_2_ that is free of defects.

These findings suggest that surface region defects introduced by La doping do not significantly alter carrier recombination at times less than 3 ns. According to Figure 4a, approximately 80% of holes have decayed during this time window, but this rapid decay is unaffected by La doping. Although there are more carrier traps in the expanded shell of the La-doped nanoparticles, their energy distribution remains the same as indicated by the single value of *E*_U_ that characterizes all samples. Consequently, recombination occurs at the same rate at times <3 ns regardless of La doping level, but the disordered shell ultimately enables more holes to create photocatalytically active hydroxyl radicals as discussed next.

### 4.4 Dye Degradation Mechanism of Undoped and La-Doped Nanoceria

One goal of this study is to elucidate the mechanism of organic molecule degradation by photoexcited nanoceria, which remains controversial in the literature. Some studies suggest that excitation generates electrons that reduce oxygen (O_2_) to superoxide ion (O_2_^·–^), which then degrades the dye.^67–69^ However, the fact that photogenerated electrons are trapped at the surface of nanoceria for hours even in air^51,60^ calls into question their ability to affect photocatalytic reactions that occur more rapidly.

To establish how La doping impacts the photocatalytic performance of our nanoceria samples under our pH 10 aqueous conditions, we first identify the reactive species responsible for dye degradation. Adding methanol, a known hole/•OH scavenger, almost fully eliminates dye degradation (Figure 6), revealing that the hole is the reactive species. The small residual degradation when methanol is present likely reflects self-degradation of the dye, which also absorbs in the UV. In aqueous conditions, photogenerated holes at the surface of metal oxide nanoparticles can oxidize water or surface hydroxides to form •OH,^34,61,70,71^ which can subsequently mineralize organic molecules.

Superoxide radicals generated when photogenerated electrons in nanoceria react with DO have been proposed to contribute to the photocatalytic degradation of dye molecules.^5,69^ However, the dye degradation kinetics of our three nanoceria samples are unchanged when DO is decreased by Ar sparging (Figure S16). Furthermore, dye degradation is unaffected by the addition of an electron scavenger (Figure S17). It is important to note that Ce^3+^ centers accumulated in the photoreduced particles react with DO as shown by the slower absorbance recovery after photoreduction when DO is removed (Figure S8). However, the slow recovery even with DO present indicates that electron transfer from Ce^3+^ centers in nanoceria to DO happens too slowly to contribute appreciably to the observed rate of dye degradation. Adding previously photoreduced particles to a RhB solution does not lead to degradation in the dark after 2 h (Figure S13b). Therefore, neither Ce^3+^ centers nor superoxide ions contribute meaningfully to dye degradation under our reaction conditions.

Adding methanol eliminates both 7HC emission and RhB degradation, indicating that •OH radicals and by extension, photogenerated holes, are the key reactive species. The results in Figure 8 strongly suggest that the enhanced performance of La-doped nanoceria in Figure 7 is due to the behavior of •OH radicals created by photoexcitation. It has been proposed that coumarin detects free •OH in solution.^71^ Coumarin does not appear to adsorb significantly to the surface of the nanoparticles at pH 10 because the absorption spectrum of the nanoceria and RhB dye dispersion is additive in the separate absorption spectra with no sign of electronic interaction between the dye and the semiconductor (Figures S12, S14). However, we cannot rule out the possibility that coumarin in solution could react with surface-trapped hydroxyl radicals that escape recombination. A further insight that does not appear to have been noted in previous studies is that the UV excitation that promotes photocatalysis also bleaches the absorption of nanoceria over time (Figure S15). This likely reduces the effectiveness of the photocatalyst, but further investigation is needed.

### 4.5 Implications of Nanoceria Recombination Dynamics

La-doped nanoceria can more efficiently produce photocatalytically active •OH radicals and accumulate more Ce^3+^ centers through photoreduction when electron–hole recombination is suppressed. Based on the unchanging decay kinetics of the hole population during our observation window of 0–3 ns, we conclude that hole scavenging reactions that shut down electron–hole recombination take place on much longer time scales. Importantly, electron–hole recombination extends over fs-ms time scales in metal oxides,^72–74^ and the recombination kinetics likely diverge in La-doped and undoped samples on longer time scales. Indeed, there is precedent for power-law recombination dynamics to exhibit complex kinetics in materials with complex energy landscapes. For example, carrier recombination in hematite is described by a power law with an exponent of 0.15 in the ps-ns window that becomes 0.47 on the μs-s time scale.^74^ In WO_3_ nanoparticles, carrier recombination proceeds at a different rate after 100 μs.^75^

Our study highlights the importance of studying recombination on the slow time scales that characterize elementary redox reactions, which take place at the catalyst surface. Many interfacial redox reactions involving metal oxides such as hole transfer to water, alcohols, SCN^–^ or electron transfer to O_2_, have been shown to span time scales from hundreds of ps to ms.^62,76–78^ The rates of these processes determine the competition between photocatalytically desirable reactions and recombination. The dynamics at these time scales can be studied with ns-TA spectroscopy, but no such study has been done on nanoceria. This study also reveals the dangers of assuming that conditions prevalent in bulk ceria such as La-induced O vacancies are relevant for few nm nanoparticles in aqueous solution. For nanoceria, photocatalytic improvements will require improved understanding of the factors governing the rate of recombination at longer time scales and the rate of the reactions that transfer surface holes into the bulk solution.

A further insight is that the conventional defect-mediated recombination framework, developed for band-like semiconductors, does not apply to polaronic metal oxides such as ceria. In band-like semiconductors with delocalized carriers, the Shockley–Read–Hall mechanism holds that defects such as oxygen vacancies act as recombination centers by providing the momentum conservation required by band-to-band recombination, thereby increasing the recombination rate.^79^ However, for a polaronic system like nanoceria, carriers are observed to be localized on the fs time scale after photogeneration, and the rate of recombination is governed either by the transport needed to bring localized electrons and holes together,^29^ or by the time needed to delocalize trapped holes at the surface by detrapping. Even with the many surface defects induced by La doping (Figure 9), charge recombination remains unchanged up to 3 ns (Figure 4), underscoring this important distinction for polaronic nanomaterials.

## 5 Conclusions

The effects of lanthanum doping on the charge carrier dynamics and photocatalytic efficiency of nanoceria were investigated using steady-state and time-resolved spectroscopy. Although further structural characterization is needed, our La-doped ceria nanoparticles synthesized at ambient temperature have approximately constant size, according to TEM measurements. Nevertheless, a greater number of Ce^3+^ ions can be accumulated by UV photoreduction compared to undoped nanoceria. In addition, ultrafast TA signals attributed to ESPs in crystalline ceria decrease in amplitude in the La-doped nanoparticles.

Both observations can be explained by a model in which La^3+^ ions are found in a highly defective, amorphous shell, which surrounds a crystalline CeO_2_ core. The shell region expands with increased La doping, increasing the maximum number of Ce^3+^ centers that can be accumulated and improving the quantum yield of generating free hydroxyl radicals in the solvent. The photocatalytic degradation of RhB is mediated by mobile hydroxyl radicals, which are formed in greater yield in the La-doped nanoparticles. Nanoceria is thus a hole-dominant photocatalyst under our conditions and probably whenever photogenerated electrons can persist in nanoceria as Ce^3+^ surface defects.

La-doped nanoparticles are better photocatalysts even though electron–hole recombination rates are unchanged during the observation window (0–3 ns) of the TA measurements. Although La doping hardly changes the movement of carriers on ultrafast time scales, it must profoundly affect the slower surface chemistry. To obtain a clearer picture, investigations into the time scale and effectiveness of •OH transfer from the nanoceria surface into the solvent are needed through study of the slower surface reactions that unfold on ns-ms time scales and beyond.

It is popular to attribute the poor performance of photocatalysts to high rates of electron–hole recombination, but recombination extends over many decades of time as carriers settle into increasingly deeper traps, making it important to probe the full range of time scales over which recombination takes place. Recombination mechanisms borrowed from traditional semiconductors are inappropriate for understanding the limits of photocatalytic performance in polaronic photocatalysts like nanoceria. More effort is needed to understand the “where, when, and how”^80^ of photogenerated carrier recombination in metal oxide photocatalysts.

## Supplementary Material



## Notes

Experimental details; characterization of nanoceria samples; analysis of XRD patterns. Reactions of nanoceria with oxidants; estimation of excitation density; data fitting for TA kinetics; Urbach energy determination estimating the number of surface shell metal atoms; additional dye-degradation kinetics; quantification of hydroxyl radicals produced by nanoceria; additional data fitting (PDF)

## Notes

C.J. and L.K. are undergraduate student authors
