# Supplementary material for: Ultrafast Charge-Carrier Dynamics and Photocatalytic Mechanisms in Lanthanum-Doped Cerium Oxide Nanoparticles
Source: J Phys Chem C Nanomater Interfaces. 2026 Sep 30;130(41):14248–59. doi: 10.1021/acs.jpcc.6c05182 (PMC13679044; doi:10.1021/acs.jpcc.6c05182)
Supplement: Supplementary file 1 [file jp6c05182_si_001.pdf]

Supporting Information

Ultrafast Charge Carrier Dynamics and Photocatalytic Mechanisms in Lanthanum-Doped Cerium Oxide Nanoparticles

Bach Pham, Claire A. Jones,[†] Luke Kline,[†] Meera Madhu, Bern Kohler*

Department of Chemistry and Biochemistry, The Ohio State University, 100 West 18th Avenue, Columbus, Ohio 43210, United States

[†]Undergraduate student authors.

*Corresponding Author: Bern Kohler, kohler@chemistry.ohio-state.edu, Tel: +1-614-688-2635

Table of Contents

S.1 Experimental details S3

S.2 Characterization of nanoceria samples S9

S.3 Analysis of XRD patterns S12

S.4 Reactions of nanoceria with oxidants S14

S.5 Estimation of excitation density S14

S.6 Data fitting for TA kinetics S17

S.7 Urbach energy determination S18

S.8 Estimating the number of surface shell metal atoms S19

S.9 Additional dye degradation kinetics S21

S.10 Quantification of hydroxyl radicals produced by nanoceria S24

S.11 Additional data fitting S26

S.12 References S27

1) Experimental details

a. Synthesis of undoped and La-doped nanoceria

Polyacrylic acid (PAA)-capped ceria nanoparticles were synthesized from cerium(III) nitrate hexahydrate ($Ce(NO_3)_3 \cdot 6H_2O$) (ThermoFisher) by slightly modifying the procedure in Asati et al.[1] Briefly, 1 mmol of $Ce(NO_3)_3 \cdot 6H_2O$ was dissolved in 5 mL of ultrapurified water from a Milli-Q system (MilliporeSigma). The solution was then added dropwise into 30 mL of 28% ammonium hydroxide (Fisher Chemical) in water under vigorous stirring, and 5 mL of a PAA (MW ~2000) aqueous solution (100 mg mL^{-1}) (Sigma Aldrich) was added to the mixture 1 min later. The mixture was stirred for 24 h, resulting in a brown dispersion and a small amount of dark brown precipitate. The mixture was then centrifuged at 4800 g for 15 minutes to isolate the supernatant, which will be referred to hereafter as the nanoceria stock dispersion. The stock dispersion has an initial pH of 12 and a cerium atom concentration of 0.025 M, or even somewhat greater due to a loss of solvent through evaporation during the 24 h reaction time. Given that the molar absorption coefficient of nanoceria is 3800 M^{-1} cm^{-1} at 290 nm,[2] the stock dispersion would produce an absorbance of nearly 100 in a 1 cm path length. This optically dense dispersion was diluted by a factor of 5 or more to produce concentrations suitable for optical measurements. Dilution was performed using either water or 80% water / 20% methanol as described below and then adjusting the pH to 10 by adding a few drops of concentrated aq. NH_4OH (28% w/w).

La-doped ceria nanoparticles were synthesized by combining sufficient $La(NO_3)_3 \cdot 6H_2O$ (ThermoFisher) with 1 mmol $Ce(NO_3)_3 \cdot 6H_2O$ to obtain a La:Ce mole ratio of 10% or 20%. The subsequent steps were identical to the procedure used for undoped nanoceria.

b. Characterization of undoped and La-doped nanoceria

Transmission electron microscopy (TEM). Bright-field TEM images were recorded using a Tecnai F20 microscope (200 kV, formerly produced by FEI). Samples for imaging were prepared by depositing a drop of the nanoceria dispersion on a 200-mesh, copper-supported carbon film (Ted Pella Inc.) and then leaving the grid to dry under air with a watch glass for a dust cover.

X-ray fluorescence (XRF). XRF spectra of nanoceria samples in the form of dry powders were taken using a Portable XRF Analyzer (Innova). Powders were obtained by precipitating nanoparticles from the nanoceria stock dispersion by adding 1 M HCl. The precipitate was collected by centrifugation (4800 g for 15 min), washed twice with water, dried under flowing air for 24 hours, and pulverized using a mortar and pestle before being analyzed.

X-ray diffraction (XRD). XRD patterns of all nanoceria samples were taken using a MiniFlex 600 X-ray diffractometer (Rigaku). The diffractometer operated at 40 kV and 15 mA with Cu Kα emission as the X-ray source. XRD patterns were recorded from the same powder samples prepared for XRF measurements.

Raman spectroscopy. Raman spectra of all nanoceria samples were recorded with excitation at 633 nm using an inVia confocal Raman microscope (Renishaw). Raman spectra were acquired from the same powder samples prepared for XRF measurements.

Dynamic light scattering (DLS). DLS measurements of all nanoceria samples were performed using a Zetasizer Pro DLS particle size analyzer (Malvern Panalytical). The instrument uses a backscatter configuration and the optimal position within the sample cell is automatically determined by the instrument to minimize effects of multiple scattering or absorbance, compared to instruments that utilize a 90° detection geometry. Data were collected and analyzed using the Zetasizer Nano software. Data quality was assessed using the derived count rate, which represents

the measured photon count rate corrected for the attenuator automatically selected by the instrument to maintain a measured count rate between (2–5) × 10^5 s^{-1}. The stock dispersion was diluted to 10% of the original concentration with water prior to the DLS measurements to minimize multiple scattering.

c. UV irradiation

For all UV irradiation experiments, e.g., photoreduction (SI section 1d), dye degradation (SI section 1f), and quantification of •OH (SI section 1g), the dispersion subjected to irradiation was first transferred into a 1.0 cm fused silica cuvette and then inserted into a Rayonet carousel photoreactor (The Southern New England Ultraviolet Company) equipped with four low-pressure Hg-vapor lamps (λ = 254 nm). Inside the photoreactor, the cuvette was placed in a cuvette holder on top of a stir plate, which was fixed at the center of the reactor to ensure that the cuvette always has the same position and orientation with respect to the lamps. During irradiation, the cuvette was capped to prevent evaporation, and the dispersion was stirred by a magnetically driven stir bar to ensure even irradiation and eliminate mass transport effects. A fan below the photoreactor ran during irradiation to prevent heat buildup.

d. Photoreduction of nanoceria samples and reactions with H_2O_2

For photoreduction experiments, the stock dispersion was diluted by adding 80% water and 20% methanol (by volume) to yield an absorbance at 290 nm ($A_{290\ nm}$) of 2.2 in a 1.0 cm path length cuvette. UV-vis spectra were recorded using a Cary 5000 UV-Vis-NIR spectrophotometer (Agilent) after every 15 min of irradiation until the absorption spectrum stopped changing. Maximum photobleaching was achieved after 60 min of irradiation in our Hg lamp photoreactor (Figure 1). Before irradiation, each sample was sparged with argon gas (flow rate 20 mL min^{-1}) for

5 min to remove dissolved oxygen (DO) using a needle inserted through a septum placed on the cuvette.

e. Transient absorption spectroscopy

Transient absorption (TA) measurements were performed using an amplified Ti:S laser system (Astrella, Coherent, Inc.), described in detail elsewhere.[3] The pump wavelength was 265 nm for all TA measurements. SI section 5 describes the experimental parameters used to generate approximately one or fewer absorbed photons per CeO_2 nanoparticle. Samples for TA measurements were prepared by diluting an aliquot from the stock dispersions using water until the absorbance at 265 nm was ~2 in a path length of 1.0 mm. The dispersions used in TA experiments were ~10 times more concentrated than ones used in photoreduction experiments (SI section 1d). Approximately 7 mL of the resulting dispersion studied were circulated through a flow cell (Harrick Scientific) fitted with Teflon spacers to obtain a 0.5 mm path length. For each TA measurement, the time delay was scanned from pre-time zero to 3.5 ns, averaging 2000 laser shots (1000 pump on/pump off pairs) per time point. Five scans were then averaged together to generate the final data set. All signals were recorded with the pump and probe polarizations set at the magic angle (54.7°).

f. Dye degradation by nanoceria samples

Samples for dye degradation experiments were prepared by diluting the stock dispersions to yield an absorbance at 290 nm ($A_{290\ \mathrm{nm}}$) of ~2.2 in a 1.0 cm path length fused silica cuvette. The same nanoceria concentrations were used in photoreduction experiments (SI section 1d). Next, 20 μL of a 4.5 mM Rhodamine B (RhB)(Sigma Aldrich) aqueous solution was added to 4 mL of the diluted nanoceria dispersion to give a RhB concentration of ~0.022 mM. The absorbance at 554

nm (A_{554}) was measured to follow the RhB degradation kinetics when the cuvette was irradiated in a Hg lamp photoreactor for up to 2 h with constant stirring.

To explore the effect of anaerobic conditions on dye degradation, the nanoceria-RhB mixtures were sparged with argon (flow rate 20 mL min^{-1}) for 5 min before irradiation. The cuvette was capped with a septum during irradiation to minimize exposure to air. To explore the effect of a hole scavenger on dye degradation, the dispersions were diluted with 80% water / 20% methanol (v/v) instead of water. To explore the effect of an electron scavenger on dye degradation, ~68 mg of $AgNO_3$ was added to the nanoceria-RhB mixture to yield 0.1 M $AgNO_3$.

To test for potential dark reactions between the reduced nanoceria and RhB, the nanoceria dispersion (A_{290} ~ 2.2) was first irradiated for 60 min in the Hg photoreactor, and then RhB was added to yield a concentration of ~0.022 mM in the mixture. This mixture was kept in the dark under stirring for 2 hours.

To monitor the concentration of RhB during degradation, the absorption coefficient of RhB at 554 nm was established beforehand. Five RhB solutions with concentrations of 0.011 mM, 0.022 mM, 0.033 mM, 0.066 mM, and 0.132 mM were prepared in pH 10 aqueous solution, and absorption spectra were measured with the UV-vis spectrometer using a 1 cm path length cuvette. The absorbance at 554 nm was plotted against concentration, and the slope of the best-fit line was used to obtain a value of 79,600 M^{-1} cm^{-1} for the molar absorption coefficient.

g. Quantification of •OH formation

Samples for •OH quantification were prepared by diluting the stock dispersions either with water or using 80% water / 20% methanol (v/v) to yield an absorbance at 290 nm ($A_{290\ nm}$) of ~0.2 in a 1.0 cm path length. Then, 8 μL of a 0.05 M coumarin solution was added to 4 mL of the diluted dispersion to yield a final coumarin concentration of ~0.1 mM. The concentration of

nanoceria was approximately 10 times smaller than in the RhB degradation experiments (SI section 1f) to avoid inner-filter effects from absorption by cerium oxide. The coumarin concentration is much higher than the concentration of photogenerated •OH allowing all hydroxyl radicals to be captured.

The mixtures were then irradiated in the Hg lamp photoreactor for 30 min under constant stirring. Emission spectra of the mixtures before and after irradiation were recorded using a QuantaMaster-8075-22 fluorimeter (Horiba) with an excitation wavelength of 370 nm, and excitation spectra were recorded with an emission wavelength of 460 nm. The photon dose absorbed by nanoceria was calculated from the nanoceria absorbance and the number of absorbed photons measured by actinometry with uridine. Full details are in SI Section 10.

To construct a calibration curve relating the fluorescence intensity of 7-hydroxycoumarin to its concentration, a series of emission and excitation spectra were recorded for five solutions of authentic 7-hydroxycoumarin (Sigma Aldrich) with different concentrations. First, 6.61 mg of 7-hydroxycoumarin was added to 150 mL of water at pH 10, adjusted by NH_4OH, to make the stock solution with a concentration of 0.27 mM. Then, serial dilution using water at pH 10 was performed to obtain five 7-hydroxycoumarin solutions with concentrations of 136 nM, 106 nM, 91 nM, 76 nM, and 60 nM. Emission spectra of these five solutions were recorded with excitation wavelength at 370 nm, and excitation spectra were recorded with emission wavelength of 455 nm.

2) Characterization of nanoceria samples

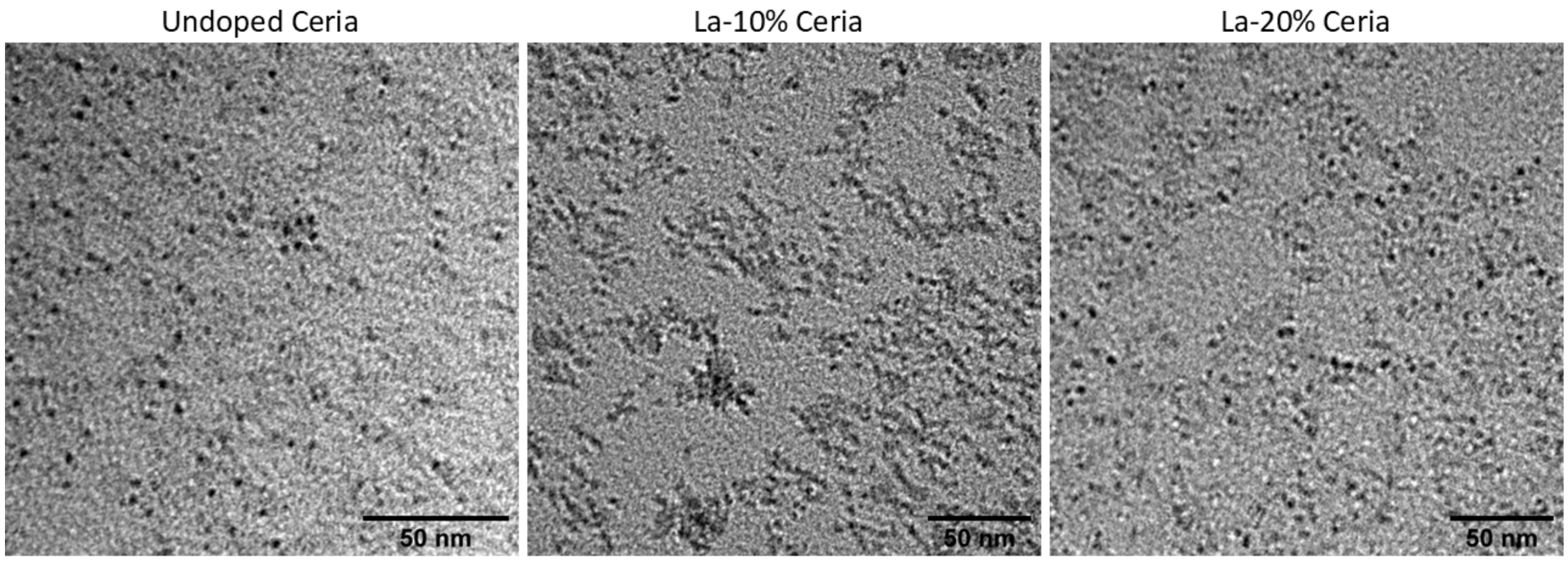

Figure S1. Bright-field TEM images of all nanoceria samples.

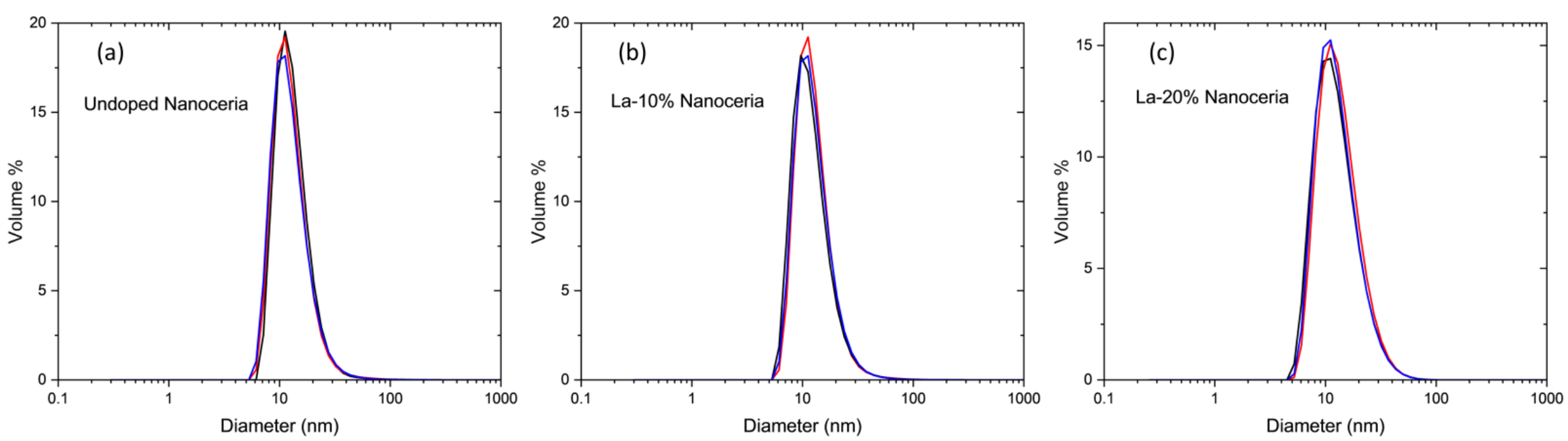

Figure S2. DLS size distribution of (a) undoped nanoceria, (b) La-10% nanoceria, and (c) La-20% nanoceria. All samples are dispersed in water. Three trials recorded from independently synthesized material are shown in each panel.

Table S1. Diameters of undoped and La-doped cerium oxide nanoparticles based on TEM and DLS measurements. Diameters obtained from TEM are averaged from 50 particles that are manually sized using the software FIJI.[4] Diameters obtained from DLS are peak values. Reported errors are 2σ.

	La-0% Nanoceria	La-10% Nanoceria	La-20% Nanoceria
TEM diameter (nm)	3.9 ± 0.6	3.9 ± 0.7	3.8 ± 0.5
DLS diameter (nm)	10.2 ± 1.4	10.3 ± 2.4	11.2 ± 1.2

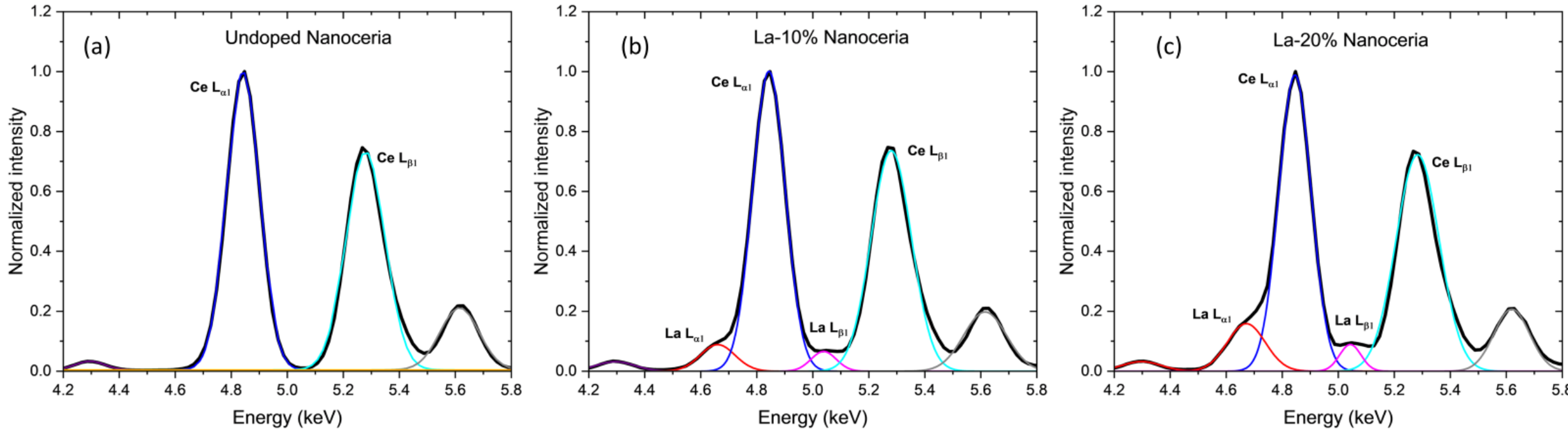

Figure S3. XRF spectra of (a) undoped nanoceria, (b) La-10% nanoceria, and (c) La-20% nanoceria. Black curves are the experimentally obtained XRF spectra, and curves in other color are Gaussian fits, with peaks chosen to be the known XRF peaks of cerium and lanthanum. All spectra are normalized to the most intense peak in each spectrum, which is the Ce $L_{\alpha 1}$ peak.

Table S2. Ratio of the integrated peak area of the La $L_{\alpha 1}$ peak compared to the integrated peak area of the Ce $L_{\alpha 1}$ peak obtained by XRF.

	La-0% Nanoceria	La-10% Nanoceria	La-20% Nanoceria
La $L_{\alpha 1}$ / Ce $L_{\alpha 1}$	0%	9.4%	19%

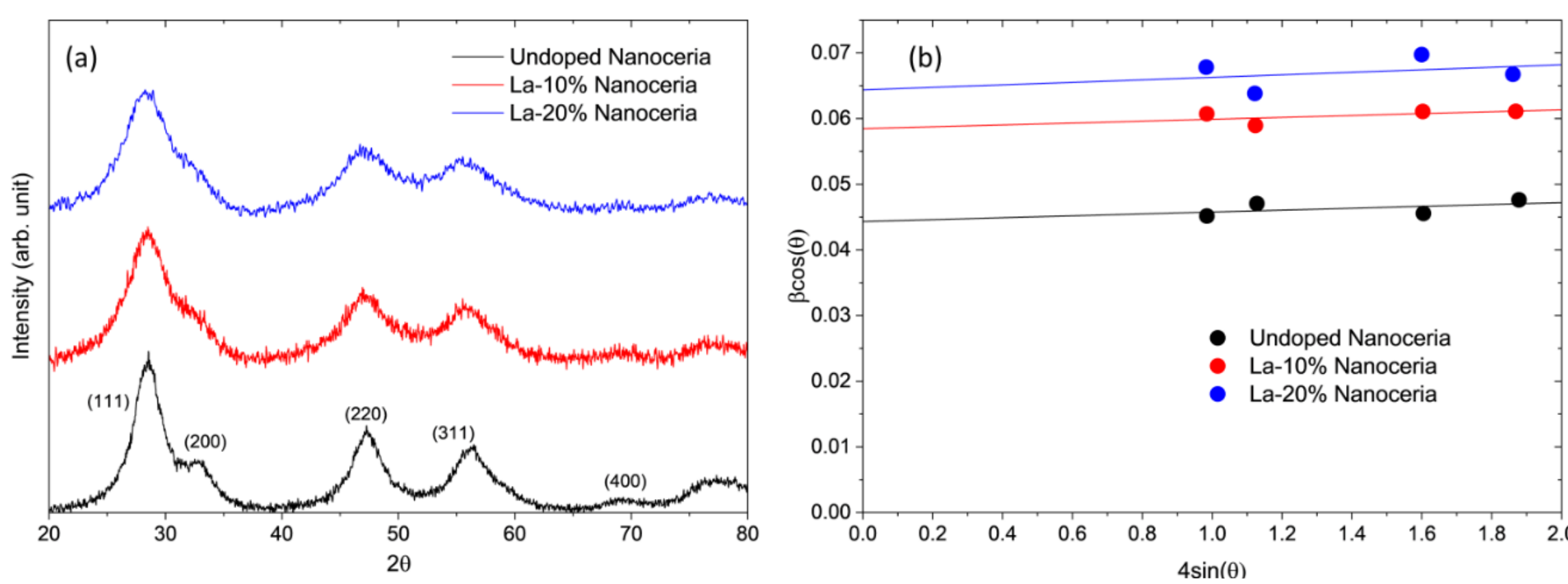

Figure S4. (a) XRD patterns of all nanoceria samples. (b) Williamson-Hall plots of all nanoceria samples using the (111), (200), (220), and (311) peaks. The slopes and y-intercepts of linear fits for each sample were used to determine the crystallite size and lattice microstrain (eq S2).

Table S3. Peak positions (θ, in radian) and FWHM (β_{hkl}, in radian) of all nanoceria samples obtained from the XRD patterns.

(hkl)	Parameters	Undoped Nanoceria	La-10% Nanoceria	La-20% Nanoceria
(111)	θ	0.2487	0.2486	0.2483
	β_{111}	0.04660	0.06266	0.06999
(200)	θ	0.2859	0.2847	0.2843
	β_{200}	0.04904	0.06144	0.06650
(220)	θ	0.4128	0.4123	0.4115
	β_{220}	0.04974	0.06667	0.07610
(311)	θ	0.4887	0.4862	0.4839
	β_{311}	0.05393	0.06911	0.07540

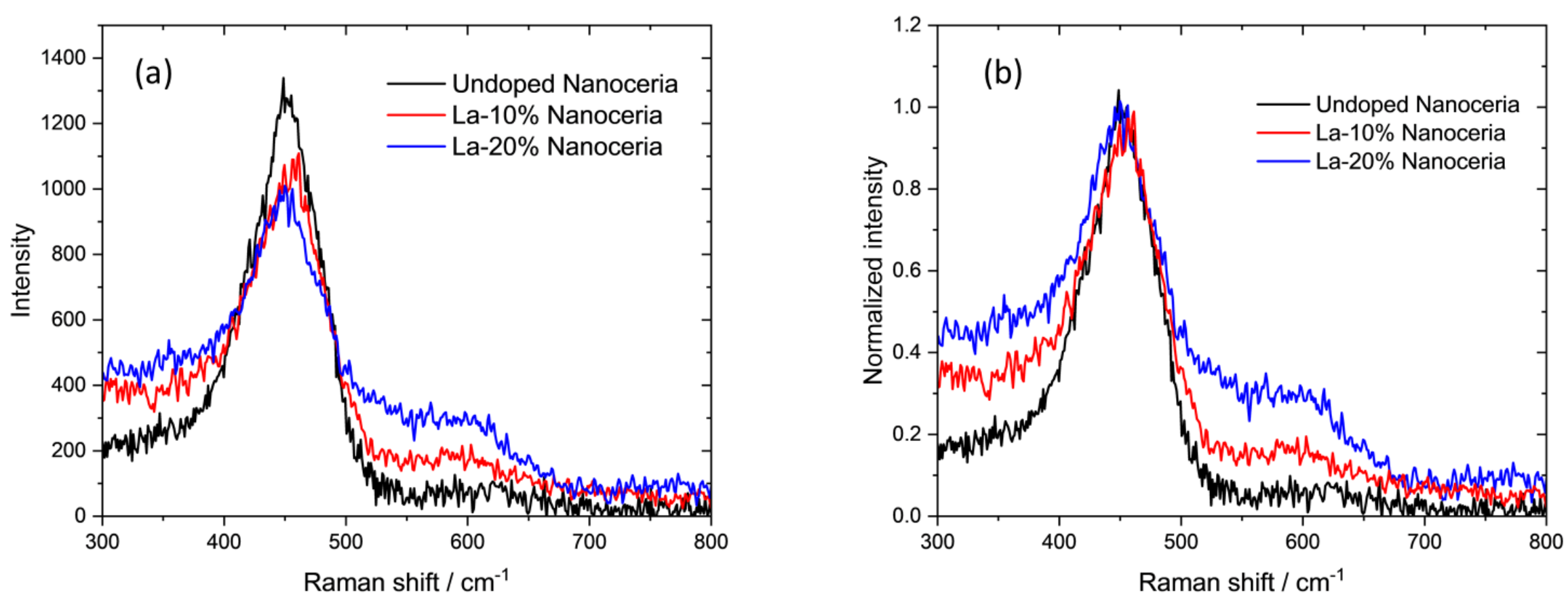

Figure S5. (a) Raw and (b) normalized Raman spectra of solid powder nanoceria samples recorded at 633 nm. Spectra in (b) are normalized at the F_{2g} peak of cerium oxide centered near 450-455 cm^{-1}.

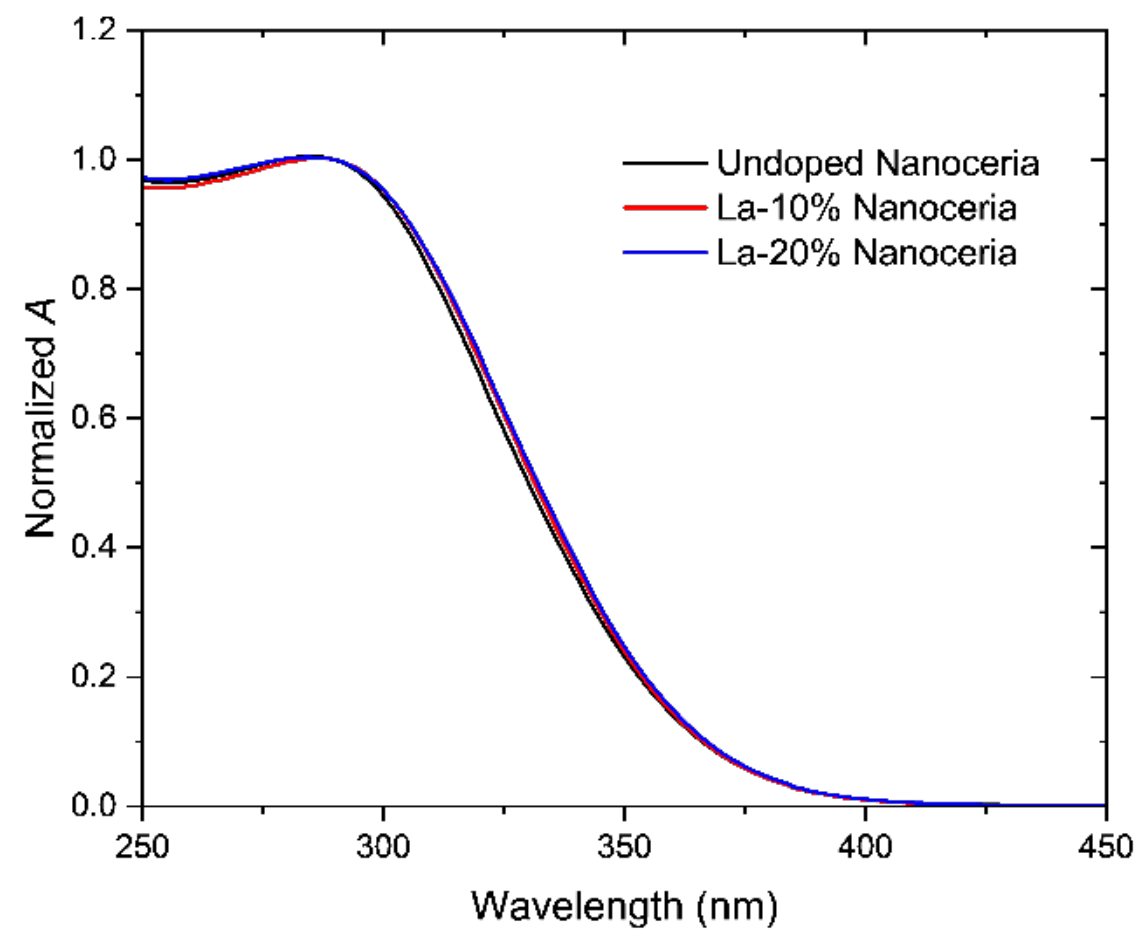

Figure S6. UV-Vis spectra of all nanoceria samples normalized at 290 nm. Methanol is present in all dispersions at 20% volume.

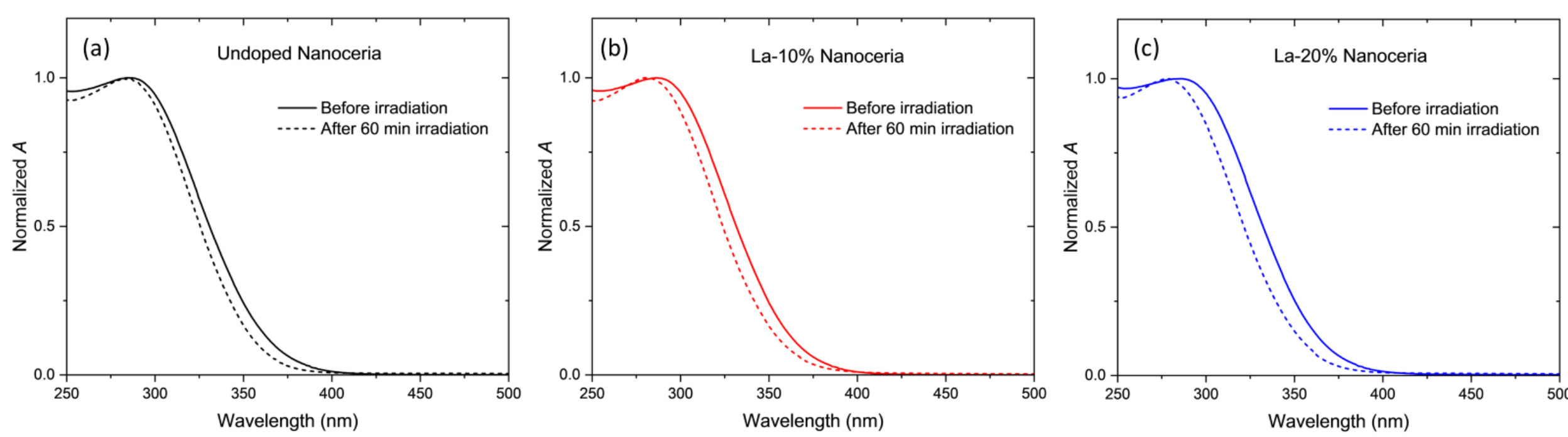

Figure S7. UV-Vis absorption spectra of **(a)** undoped nanoceria, **(b)** La-10% nanoceria, and **(c)** La-20% nanoceria dispersed in water at pH 10 before and after irradiation with UV light (λ = 254 nm) for 60 min, all normalized to unity at the maximum absorbance of the spectra. The original spectra before normalization are shown in Figure 1.

3) Analysis of XRD patterns

The XRD patterns in Figure S4a were analyzed to obtain the lattice parameter, crystallite size (diameter), and lattice microstrain. Eq S1 was used to calculate the lattice parameter, a, using,

$$a = \frac{\lambda}{2 \sin \theta} \sqrt{h^2 + k^2 + l^2} \quad \text{(S1)}$$

where λ is the Cu Kα wavelength of 0.154 nm, θ is the diffraction angle in radians, and h, k, and l are the Miller indices, all with the value of 1 as the most prominent (111) peak was used. The crystallite sizes and lattice microstrain were calculated using the Williamson-Hall plot (Figure S4b). The Williamson-Hall plot takes into account the broadening effects from both crystallite sizes and microstrain,[5]

$$\beta_{hkl} \cos\theta = \left(\frac{s\lambda}{D}\right) + 4\varepsilon \sin\theta \tag{S2}$$

where β_{hkl} is the FWHM of the corresponding (hkl) peak, s is the shape factor, taken to be 0.94 for a spherical shape, D is the crystallite diameter, and ε is the lattice microstrain. By plotting $\beta_{hkl} \cos\theta$ against $4\sin\theta$ using the (111), (200), (220), and (311) peaks (Figure S4b), the y-intercept of the linear fit was used to estimate D, and the slope of the fit was used to obtain ε. The lattice parameter, crystallite diameter, and microstrain are listed for each sample in Table S4.

Table S4. Lattice parameters, crystallite sizes, and microstrain of all nanoceria samples obtained from the XRD patterns. The data is averaged from 3 separate acquisitions of samples from 3 different synthesis batches. All uncertainties are 2σ.

Samples	Lattice parameter (Å)	Crystallite Diameter (nm)	Lattice microstrain
Undoped Nanoceria	5.420 ± 0.001	3.2 ± 0.4	0.0014 ± 0.0009
La-10% Nanoceria	5.422 ± 0.002	2.7 ± 0.3	0.0014 ± 0.0008
La-20% Nanoceria	5.429 ± 0.002	2.4 ± 0.3	0.0016 ± 0.0009

4) Reactions of nanoceria with oxidants

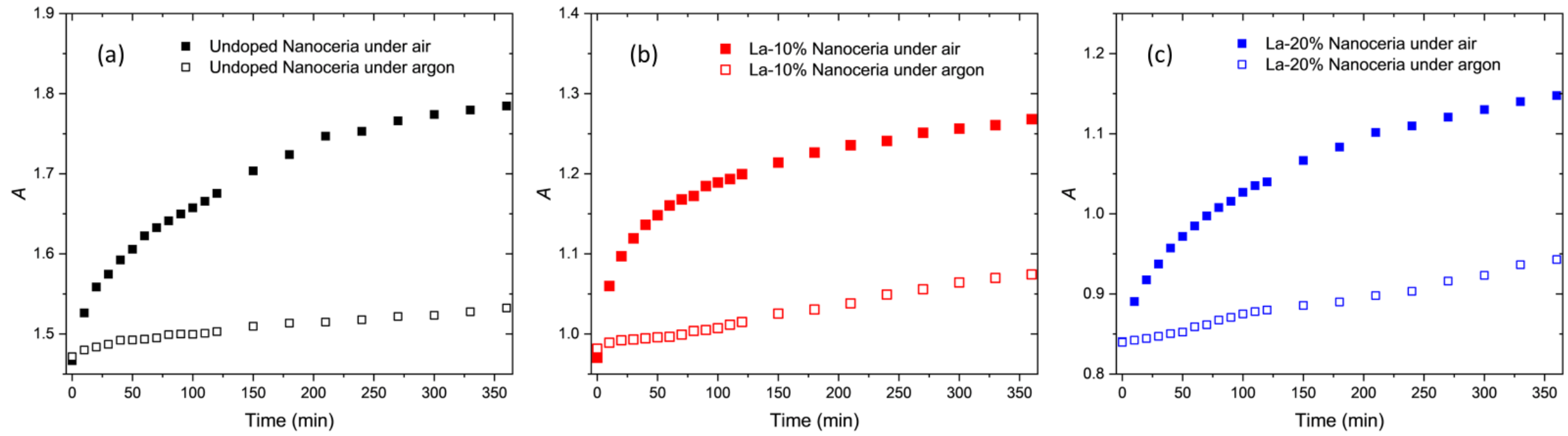

Figure S8. Spontaneous re-oxidation of the photoreduced samples monitored by the absorbance at 290 nm of (a) undoped nanoceria, (b) La-10% ceria, and (c) La-20% ceria with actively bubbled air or argon (flow rate of 20 mL min^{-1}).

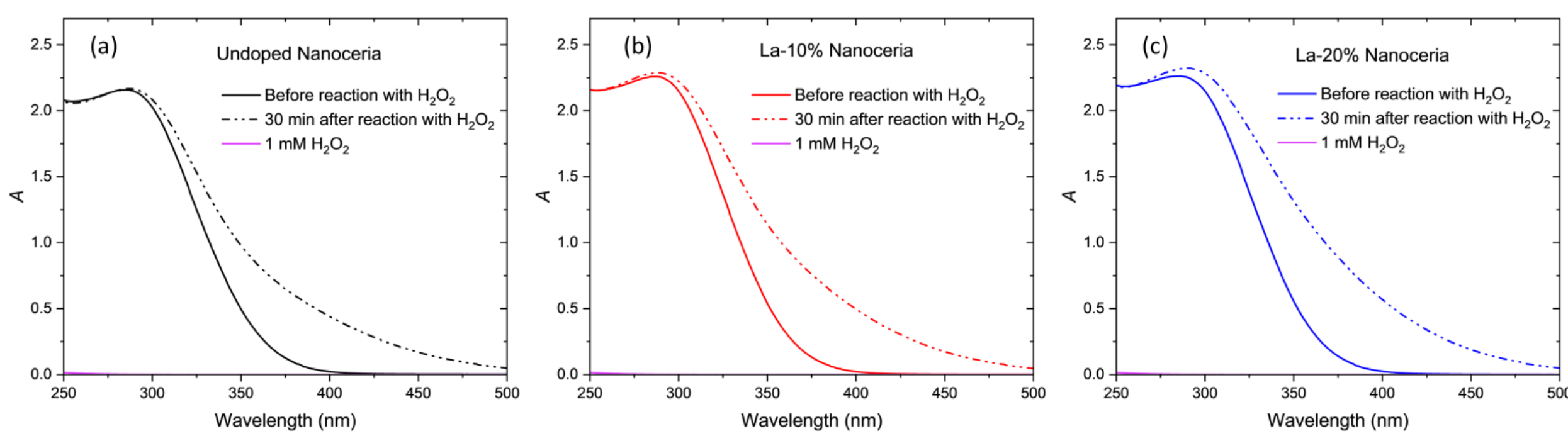

Figure S9. Absorption spectra of aqueous dispersions of (a) undoped ceria, (b) La-10% ceria, and (c) La-20% ceria before and 30 min after reaction with 1 mM H_2O_2. Absorption by 1 mM H_2O_2 in water is negligible in this window (solid magenta curves).

5) Estimation of excitation density

The average number of excited states formed by the pump pulse per nanoparticle was estimated following the method described in our previous work[6] and reiterated in this section. The average number of excited states per unit volume of the sample, ρ_{exc}, created by a pump pulse of wavelength, λ, in a sample with path length L is given by,

$$\rho_{\mathrm{exc}} = \frac{\lambda F_0\left(1-10^{-A}\right)}{hcL}, \tag{S3}$$

where A is the absorbance of the sample at the pump wavelength and F_0 is the peak energy fluence (energy per unit area) of the pump pulse. The intensity of the pump and probe lasers beams was assumed to vary in a plane transverse to the beam propagation direction as $e^{-2\left(\frac{r}{w}\right)^2}$ (Gaussian beams), where r measures the distance from the center of the beam and w is the 1/e^2 beam radius. Using the razor blade technique,[7] w was measured to be 830 mm for the 265 nm pump beam for experiments shown in Figures 2, 3, and 4. The probe was measured to be ≤ 250 mm across the working range.

For a beam with a Gaussian spatial profile, the peak fluence is given by,

$$F_0 = \frac{2E_\mathrm{p}}{\pi w^2}, \tag{S4}$$

where E_p is the pump pulse energy. The pump pulse energy was obtained to be 2.28 μJ by measuring the power in the pump beam immediately before the sample cuvette and dividing by the laser repetition rate (1 kHz). No adjustment was made for partial reflection (Fresnel losses) of the pump pulse by the front window of the cuvette as such losses are estimated to be < 5%. The average number of excitations per nanoparticle, N_p, was calculated by dividing the number of excitations per unit volume in the sample (eq S3) by the number of nanoparticles per unit volume, ρ_p,

$$N_\mathrm{p} = \frac{\rho_\mathrm{exc}}{\rho_\mathrm{p}}. \tag{S5}$$

The sample absorbance A was used to calculate ρ_p,

$$\rho_\mathrm{p} = \frac{AN_\mathrm{A}}{\epsilon L N_\mathrm{Ce}}, \tag{S6}$$

where N_A is Avogadro's constant, ϵ is the absorption coefficient for cerium oxide per mole of cerium atoms, and N_Ce is the number of cerium atoms in a single nanoparticle. The latter quantity was estimated from the volume of the nanoparticle estimated as a sphere of diameter d divided by the volume of the cubic unit cell of crystalline cerium oxide and multiplying the result by 4, the number of Ce atoms per unit cell,

$$N_\mathrm{Ce} = \frac{2}{3}\pi\left(\frac{d}{a}\right)^3 \tag{S7}$$

where a is the lattice parameter for bulk cerium oxide (0.541 nm).[8] For La-doped nanoceria, the quantity estimated from eq S7 is the total number of metal atoms, and the number of Ce atoms is calculated accordingly based on the La:Ce ratio obtained from XRF (Table S2). Combining eqs S3, S5, and S6 yields,

$$N_{\mathrm{p}} = \frac{\lambda F_0 (1 - 10^{-A}) \epsilon N_{\mathrm{Ce}}}{hcAN_A} \tag{S8}$$

For the pump wavelength of 265 nm (4.68 eV) and a path length L of 0.5 mm, the absorbance of undoped ceria, La-10% ceria, and La-20% ceria is listed in Table S5. The molar absorption coefficient ϵ at 265 nm for cerium oxide has been determined to be independent of nanoparticle size and is estimated to be 3600 ± 400 L mol^{-1} cm^{-1} (2σ), where concentration is measured in moles of Ce atoms.[2] Table S5 also lists the calculated parameters for all nanoceria samples.

Table S5. Average particle volume and number of Ce atoms per particle. Sample absorbance, pump fluence, and excitation density used in the TA experiments reported in Figures 2 – 4 (main text). Absorbance was originally measured in 1 mm path length, so the measured values were halved to reflect absorbance with path length 0.5 mm. In La-doped nanoceria, the number of Ce atoms were calculated from the total metal atoms in a particle and the La-to-Ce ratio. All uncertainties are 2σ.

	Undoped Nanoceria	La-10% Nanoceria	La-20% Nanoceria
Particle volume (nm^3)	31 ± 12	31 ± 14	29 ± 9
Ce atoms per particle	780 ± 300	710 ± 330	610 ± 190
Absorbance at 265 nm	0.87	0.83	0.81
Fluence (μJ cm^{-2})	210 ± 15	240 ± 15	280 ± 15
Excitations per particle	1.3 ± 0.5	1.4 ± 0.6	1.3 ± 0.4

6) Data fitting for TA kinetics

The decay traces of the UV band (Figure 3a) were fit to eq 1 in the main text using OriginPro software using the Levenberg-Marquardt algorithm. Convolution between the fitting function and the instrument response function was not performed. Effects due to finite time resolution should be minor because all fits excluded data points at delay times < 0.3 ps. The fitting parameters and uncertainties in Table S6 were determined by averaging fitting results from 5 independent trials. The average decay time $\langle\tau\rangle$ of the stretched exponential function is calculated as $\langle\tau\rangle = \frac{\tau}{\beta}\Gamma\left(\frac{1}{\beta}\right)$.[9]

Table S6. Best-fit parameters for the UV band kinetics. Kinetic traces were fit to a stretched exponential function, $A_0 e^{-\left(\frac{t}{\tau}\right)^{\beta}}$ (eq 1 in the main text). Reported uncertainties are 2σ.

	Undoped Nanoceria	La-10% Nanoceria	La-20% Nanoceria
A_0	4.0 ± 0.3	3.0 ± 0.3	2.5 ± 0.4
τ / ps	7.0 ± 0.5	6.0 ± 0.3	4.5 ± 0.3
β	0.40 ± 0.03	0.43 ± 0.02	0.40 ± 0.02
$\langle\tau\rangle$ / ps	24 ± 4	16 ± 4	14 ± 3

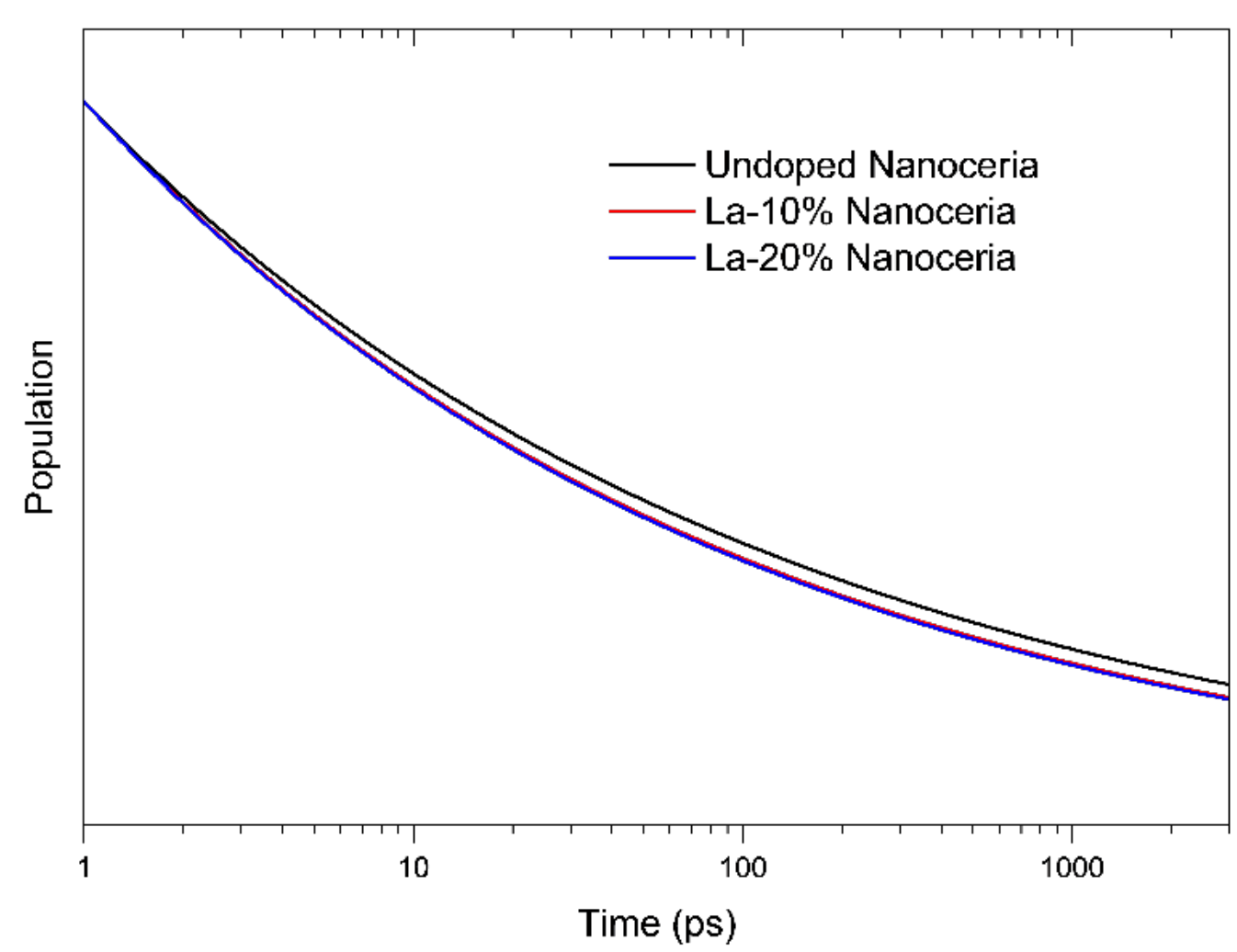

Figure S10. Surface recombination kinetics obtained from the trapping-detrapping model (eq 2 in the main text) for all nanoceria samples. The Urbach energies used in the model are obtained from the UV-vis absorption spectra as described in SI Section 7.

7) Urbach energy determination

The Urbach energy was determined by fitting the exponential tail of the UV-vis spectrum of each sample to eq S9,[10]

$$\alpha = \alpha_0 \exp\left(\frac{h\nu - E_0}{E_U}\right) \quad \text{(S9)}$$

where α is the absorption coefficient, E_U is the Urbach energy, E_0 is the band gap energy, and $h\nu$ is the photon energy. When $\ln(\alpha)$ is plotted against $h\nu$, the Urbach region is the linear portion of the graph, and the slope of the linear fit is $\frac{1}{E_U}$,

$$\ln(\alpha) = \left(\frac{1}{E_U}\right) h\nu + \ln(\alpha_0) - \frac{E_0}{E_U} \quad \text{(S10)}$$

Figure S11 shows the $\ln(\alpha)$ vs $h\nu$ plots for all nanoceria samples. Similar sigmoidal graphs have been observed for ZnO nanoparticles.[11] Also shown in Figure S11 are best-fit lines to the linear or Urbach region of the graph , which falls below the bandgap energy of bulk CeO_2 of 4.0 eV.[12] The fits were performed using only data points with energies between 3.0 eV and 3.35 eV. The value of E_U determined from the inverse slope of the fit curve is shown in each graph.

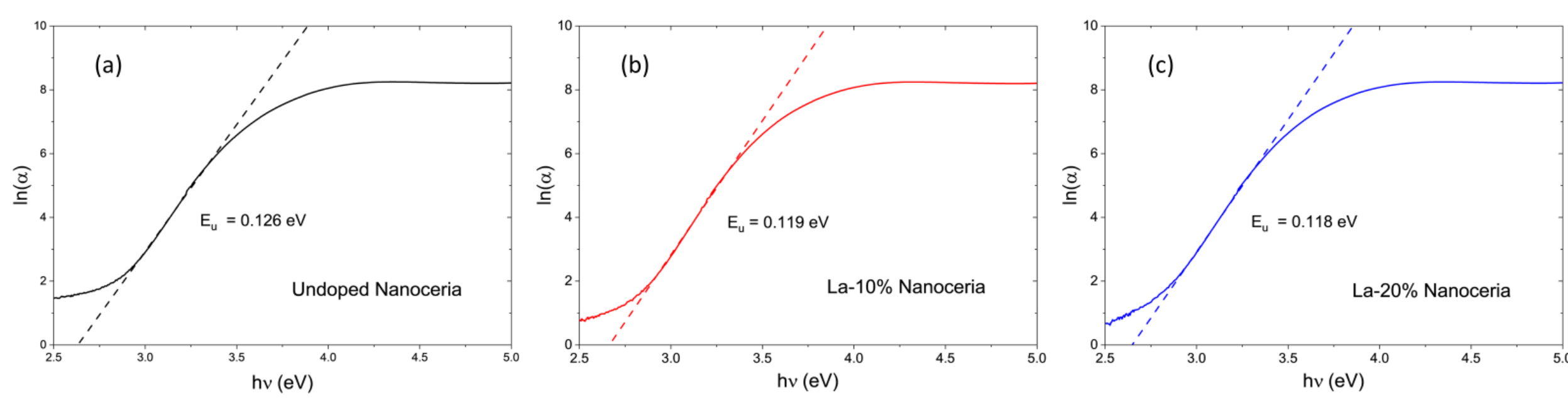

Figure S11. Graphs of the natural logarithm of the measured absorbance, $\ln(\alpha)$, vs $h\nu$ for all nanoceria samples. The slopes of the linear fits through the Urbach region (dashed line) were used to determine the Urbach energies, E_U, listed in the graphs.

8) Estimating the number of surface shell metal atoms

The number of metal atoms in the shell region of the various nanoparticles was calculated from the volume of the surface region, which is defined to be the difference between the particle volume, V_{particle}, calculated from the TEM diameter (Table S1), and the core volume, V_{core}, is calculated from r_{core} obtained from the XRD crystalline diameter (Table S4). Unlike in our previous study, we did not assume a surface thickness in this study; instead, the surface thickness was determined from the TEM sizes and XRD sizes in this work.

Given that there are four metal ions in each cubic unit cell of the fluorite crystal structure, the number of metal atoms, N_{metal} in a given volume, V, is calculated using,

$$N_{\text{metal}} = \frac{4V}{a^3} \,. \tag{S11}$$

using the lattice parameter a listed in Table S4 for each sample. For simplicity, we assume that the number density of metal atoms in the shell and in the core is the same. Using eq S11, we estimate the number of metal atoms in the core, N_{core}, and in the entire particle, N_{tot}, using the corresponding V_{core} and V_{particle}.

The number of shell metal atoms, N_{shell} is estimated by,

$$N_{\text{shell}} = N_{\text{tot}} - N_{\text{core}} \tag{S12}$$

Based on the La/Ce ratio determined by XRF (Table S2), we estimate the number of La atoms, $N_{\text{La,tot}}$, and Ce atoms, $N_{\text{Ce,tot}}$ in each particle. Assuming that all La atoms are in the surface shell, we can estimate the number of shell Ce atoms, $N_{\text{Ce,shell}}$,

$$N_{\text{Ce,shell}} = N_{\text{shell}} - N_{\text{La,tot}} \tag{S13}$$

The results are listed in Table S7.

Table S7. Particle volume V_P, volume of the crystalline core V_C, total number of metal atoms in a particle N_{tot}, number of Ce atoms in a particle $N_{Ce,tot}$, number of La atoms in a particle $N_{La,tot}$, number of metal atoms in the crystalline core N_{core}, number of metal atoms in the surface shell N_{shell}, number of Ce atoms in the surface shell $N_{Ce,shell}$, ratio of La to Ce on the surface La/Ce_{shell}, and percentage of surface Ce to total Ce in a particle $N_{Ce,shell}$ / $N_{Ce,tot}$ (%). The quantity x, appeared in Figure 9 of the main text, describes the composition of the surface shell upon La doping and is calculated from La/Ce_{shell}.

**Samples**	**Undoped Nanoceria**	**La-10% Nanoceria**	**La-20% Nanoceria**
V_P / nm^3	31 ± 12	31 ± 14	29 ± 9
V_C / nm^3	17 ± 6	11 ± 3	7.6 ± 2.4
N_{tot}	780 ± 300	780 ± 360	730 ± 230
$N_{Ce,tot}$	780 ± 300	710 ± 330	610 ± 190
$N_{La,tot}$	0 ± 0	70 ± 30	120 ± 40
N_{core}	410 ± 150	270 ± 90	190 ± 60
N_{shell}	370 ± 340	510 ± 370	540 ± 240
$N_{Ce,shell}$	370 ± 340	440 ± 370	420 ± 240
La/Ce_{shell}	0	0.16	0.29
x	0	0.14	0.23
$N_{Ce,shell}$ / $N_{Ce,tot}$ (%)	47%	61%	68%

9) Additional dye degradation kinetics

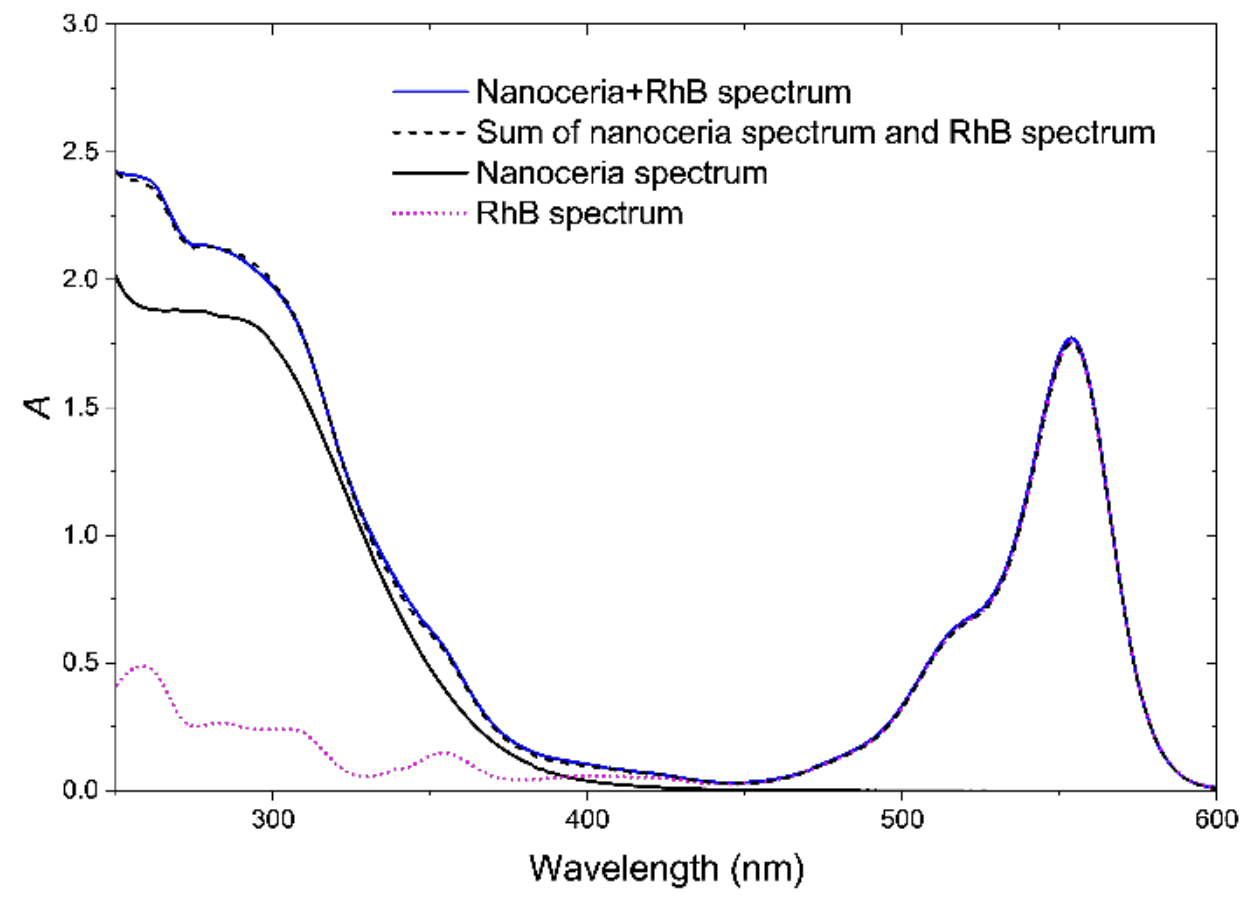

Figure S12. Absorption spectra of the nanoceria+RhB mixture, nanoceria only, and RhB only. The sum of the nanoceria only and RhB only spectra (solid black curve + dashed magenta curve) is shown for comparison.

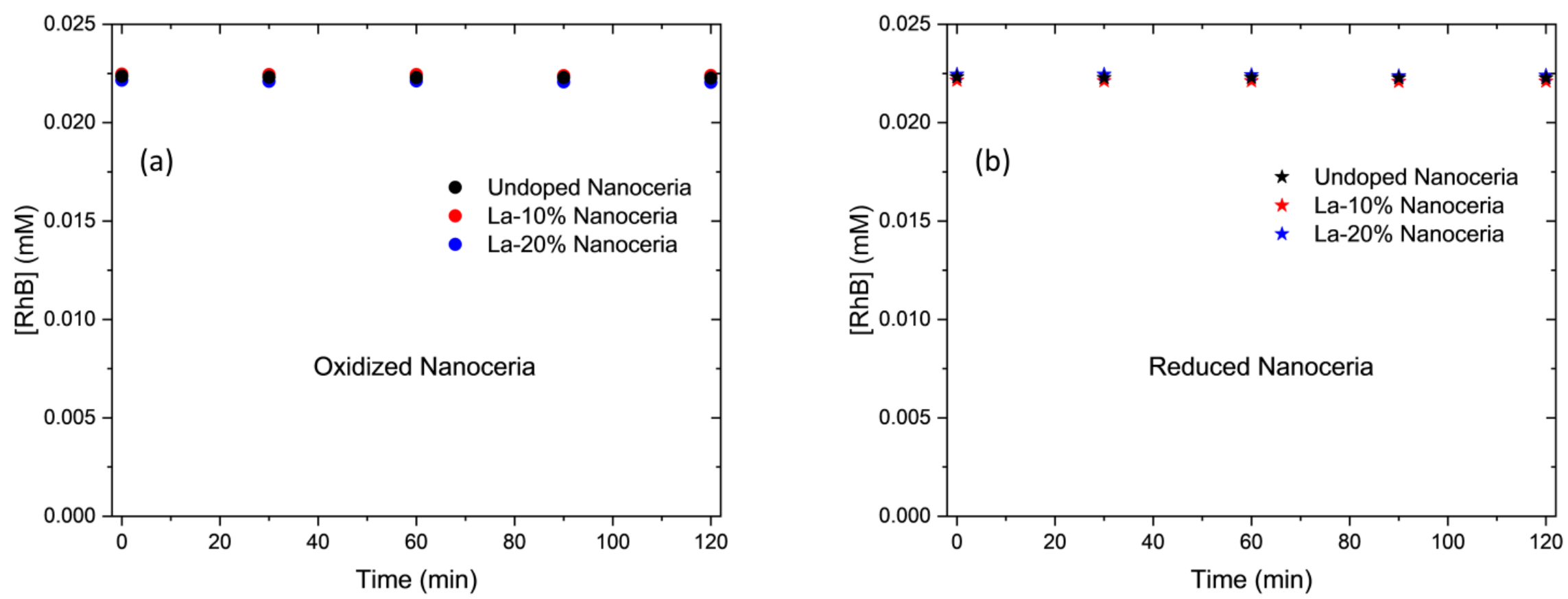

Figure S13. Concentration of RhB assayed by its absorbance at 554 nm as a function of incubation time in the dark after adding (a) oxidized or (b) photoreduced nanoceria samples. These measurements were conducted on aerated dispersions. The photoreduced nanoceria samples were produced by irradiating (λ = 254 nm) the oxidized nanoceria samples for 60 min. The initial concentration of RhB dye in all trials is 0.022 mM. Subsequent RhB concentrations were determined using the absorption coefficient of the dye at 554 nm (79,600 M^{-1} cm^{-1}). The unchanging concentration of RhB rules out a significant dark reaction.

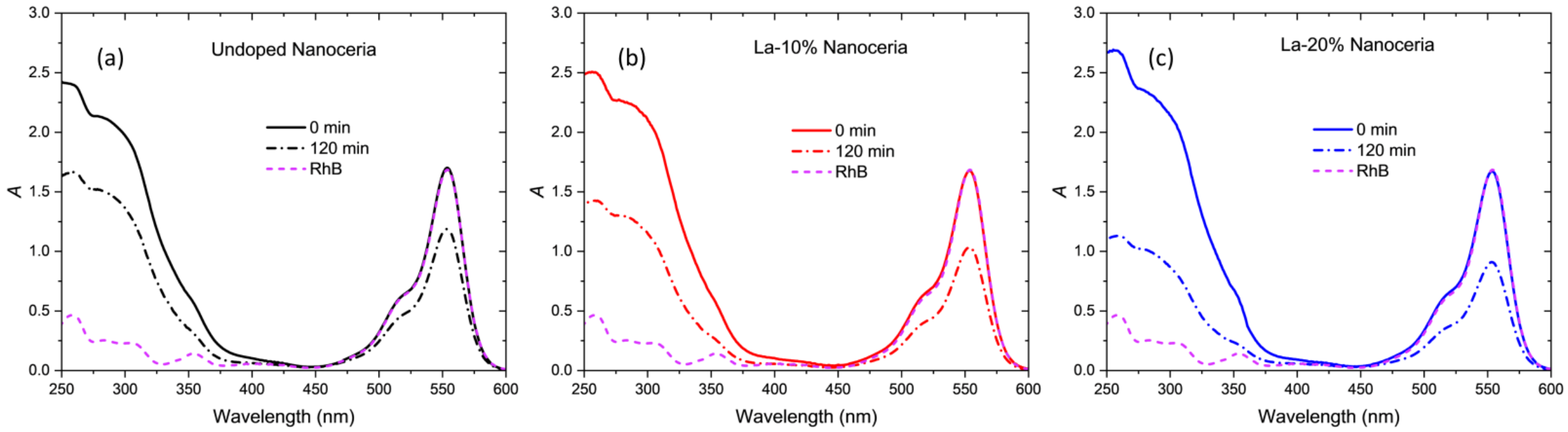

Figure S14. Absorption spectra of the nanoceria and RhB dye mixtures for (a) undoped nanoceria, (b) La-10% nanoceria, and (c) La-20% nanoceria recorded before and after dye degradation by UV irradiation (λ = 254 nm) for 120 minutes. The initial concentration of the nanoceria and RhB dye is ~0.5 mM and 0.022 mM, respectively. The absorption spectrum of a separate 0.022 mM aqueous solution of RhB without nanoceria is shown by the dashed magenta curve in each panel.

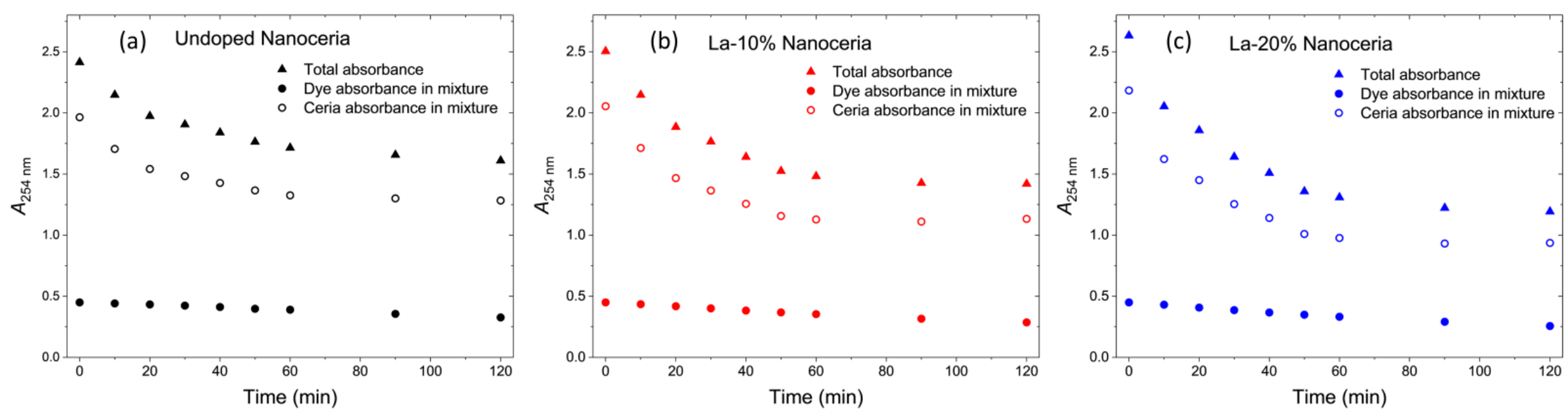

Figure S15. The measured absorbance of the nanoceria+RhB mixtures (triangle markers), RhB in the mixture (filled circle markers), and nanoceria in the mixture (open circle markers), all at 254 nm, during dye degradation for (a) undoped nanoceria, (b) La-10% nanoceria, and (c) La-20% nanoceria. The absorbance values for RhB at 254 nm at any time during irradiation were determined by scaling the absorption spectrum of the dye to match the visible wavelength absorption peak in the mixture at that time. The absorbance values for nanoceria at 254 nm were calculated as the measured absorbance of the mixture at 254 nm minus the absorbance of the dye at 254 nm.

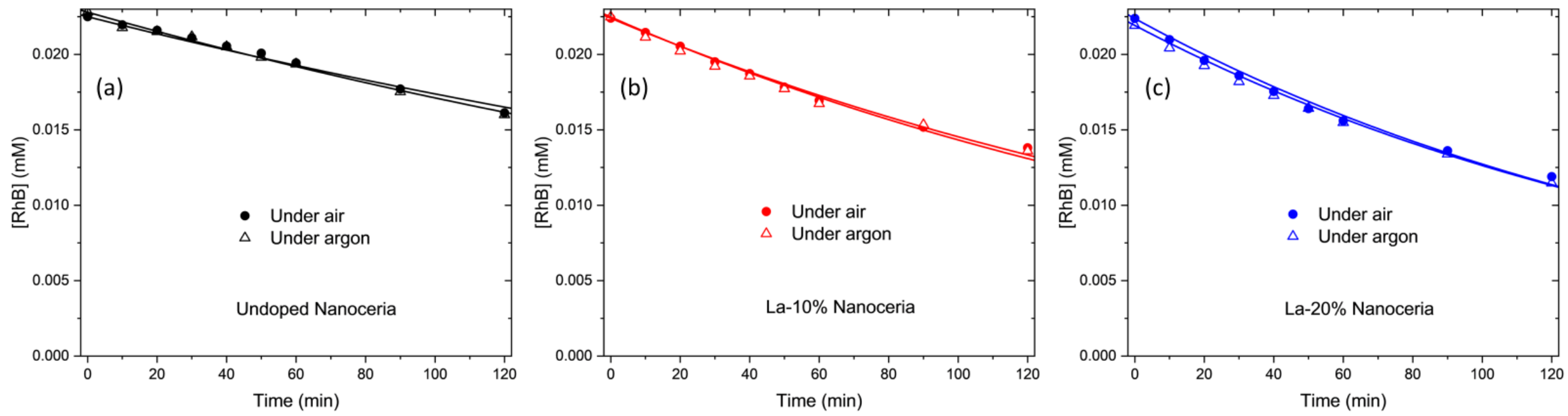

Figure S16. Dye degradation kinetics tracked by the absorbance of RhB at 554 nm of (a) undoped nanoceria, (b) La-10% nanoceria, and (c) La-20% nanoceria samples under UV irradiation (λ = 254 nm) with and without the removal of air by Ar bubbling. All samples are in water at pH 10. Solid curves are fits to a first-order kinetic model.

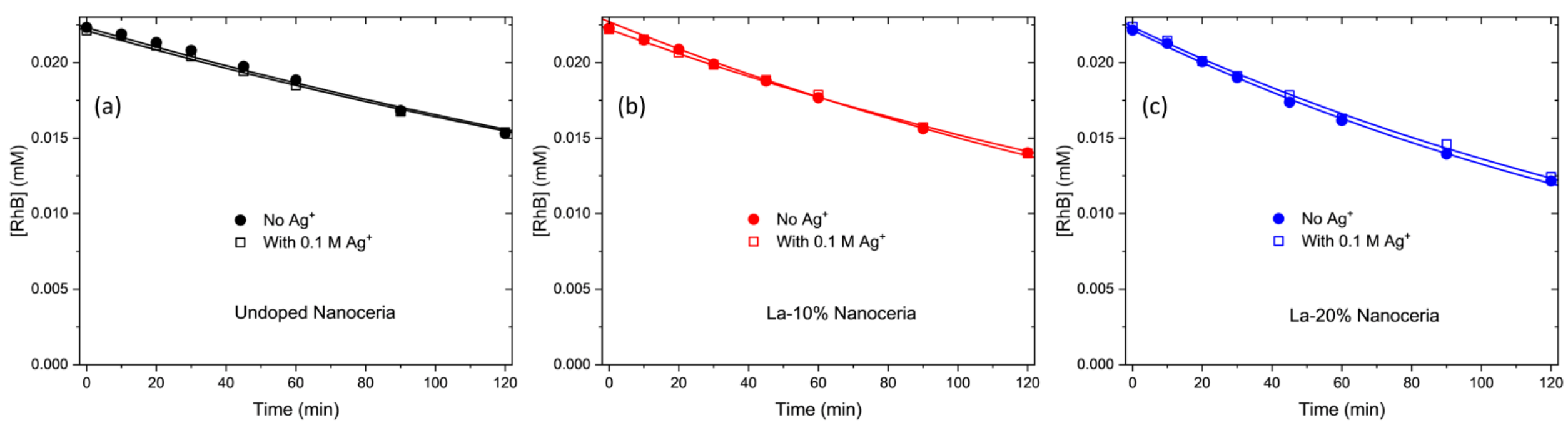

Figure S17. Dye degradation kinetics tracked by the absorbance of RhB at 554 nm of (a) undoped nanoceria, (b) La-10% nanoceria, and (c) La-20% nanoceria samples dispersed in water at pH 10 under UV irradiation (λ = 254 nm) with and without 0.1 M $AgNO_3$, all with air present. Solid curves are fits to a first-order kinetic model.

10) Quantification of hydroxyl radicals produced by La-doped nanoceria

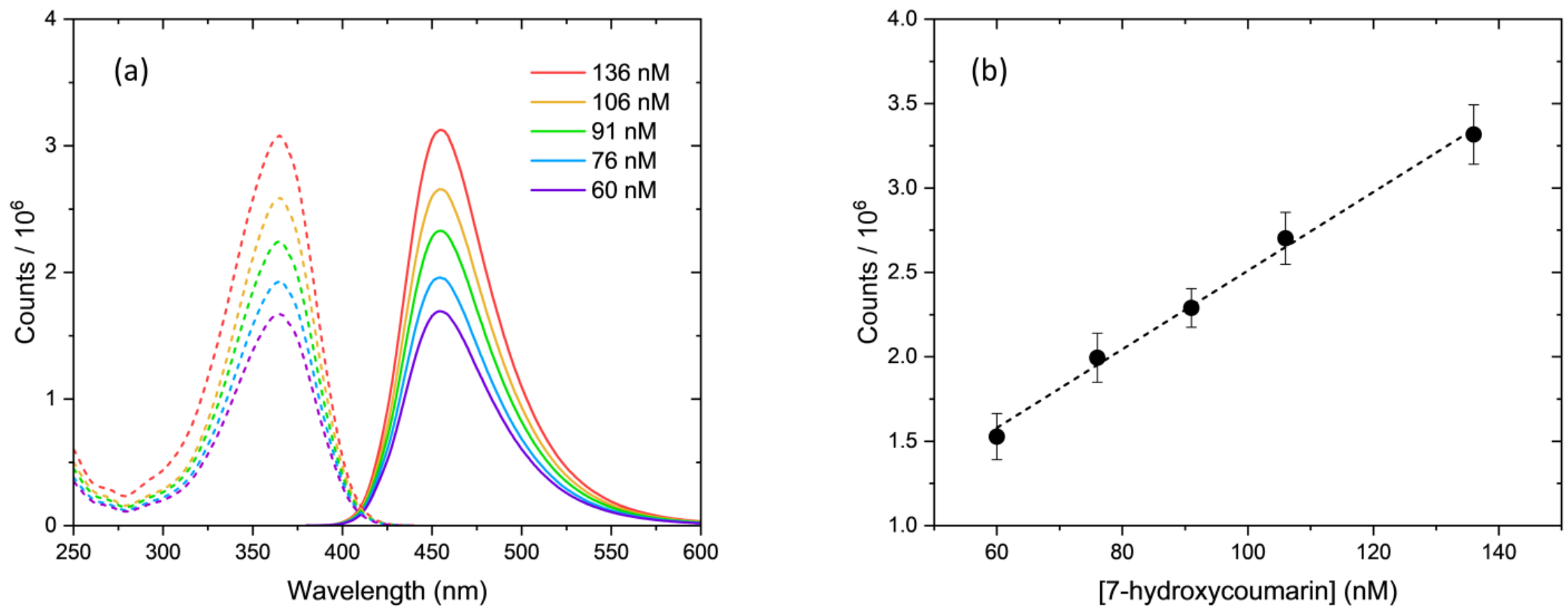

Figure S18. (a) Emission (solid curves) and excitation (dashed curves) spectra of solutions of 7-hydroxycoumarin in water at pH 10 at the concentrations indicated. Emission spectra are recorded at excitation wavelength 370 nm. Excitation spectra are recorded at emission wavelength 455 nm. (b) Maximum emission intensity (markers) of 7-hydroxycoumarin solutions in water at pH 10 as a function of their concentration. The best-fit line (dashed curve) has a slope of 23,300 nM^{-1} and y-intercept of 1.8×10^5. Error bars are 2σ uncertainties estimated from 3 independent trials.

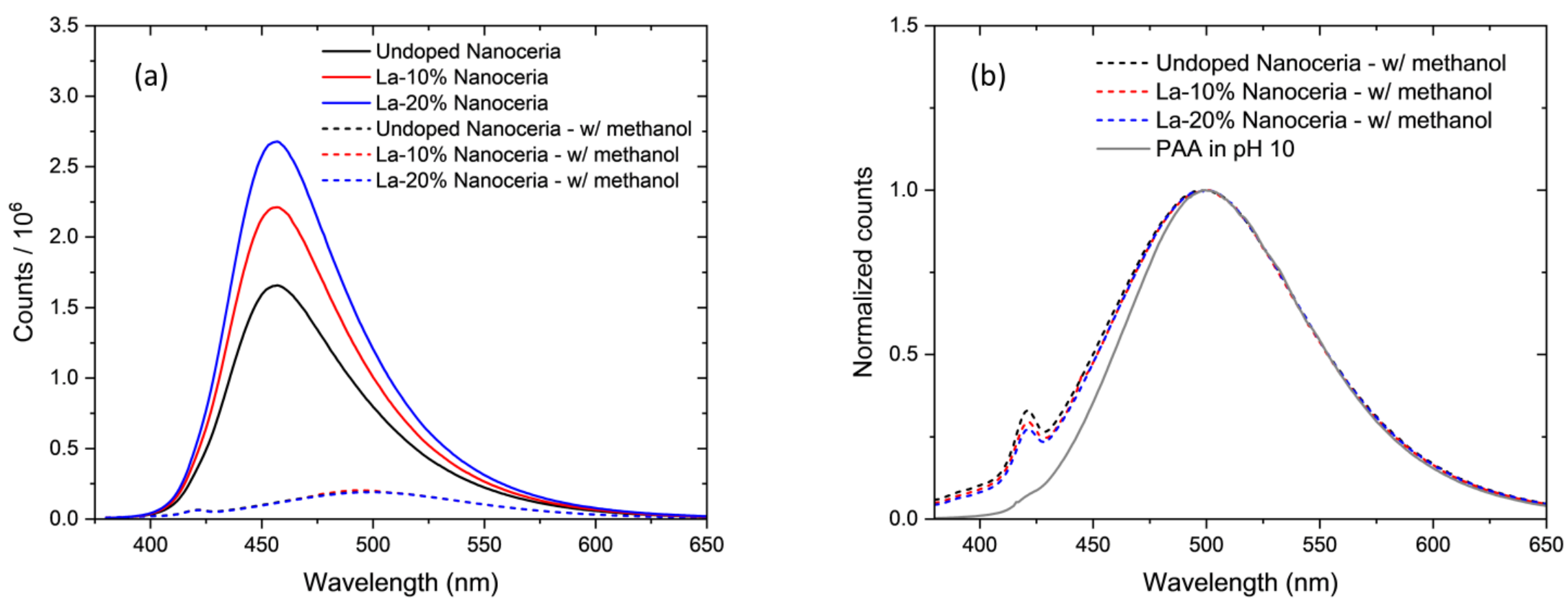

Figure S19. (a) Emission spectra 30 min after UV irradiation (λ = 254 nm) of all nanoceria samples in pH 10 aqueous solution in the presence of 0.1 mM coumarin (three solid curves) and in 80% / 20% (v/v%) water-methanol mixture (dashed curves). The emission excitation wavelength (λ_{em}) is 370 nm. (b) The emission spectra shown by dashed lines in (a) compared to the emission spectrum of PAA (5 mg mL^{-1}) after 30 min of UV irradiation (λ = 254 nm) in aqueous solution in pH 10 with neither nanoceria nor methanol present. All emission spectra have been normalized at 500 nm and $\lambda_{em} = 370$ nm.

Actinometry was performed to quantify the moles of absorbed photons per unit volume inside the cuvette during irradiation in the photoreactor. A 4.0 mL uridine solution with an absorbance at 254 nm of approximately ~3 was transferred to a 1-cm path length cuvette and irradiated in the photoreactor for 10 min under stirring. The absorption spectrum of an aqueous solution of uridine was recorded periodically (Figure S20a). The absorbance at 262 nm over time was used to graph ln([Uridine]$_t$ / [Uridine]$_0$) vs. time to obtain a first-order rate constant of 0.0928 min^{-1}. The initial reaction rate obtained from the rate constant was 2.87×10^{-5} M min^{-1}, and the volume-averaged rate of photon absorption, q_p, was determined using,[13]

$$q_p = -\frac{\frac{d\,[\text{Uridine}]}{dt}}{\Phi} \qquad \text{(S14)}$$

where Φ is the quantum yield of uridine under 254 nm irradiation and has a value of 0.019.[14] The absorbed photon rate was calculated to be 0.0015 M min^{-1}. For 30 min, the absorbed photon dose by uridine was 0.045 M. Given that the absorbance of uridine at 254 nm in the actinometry measurements is 3.15 and the absorbance of nanoceria in the •OH probing experiments at 254 nm is 0.20, the absorbed photon dose by nanoceria was estimated by multiplying 0.045 M by $\frac{1-10^{-0.20}}{1-10^{-3.15}}$, yielding an absorbed photon dose of 0.017 M. The quantum yield of •OH production listed in Table 1 in the main text was calculated by dividing [•OH]$_{total}$ by this value.

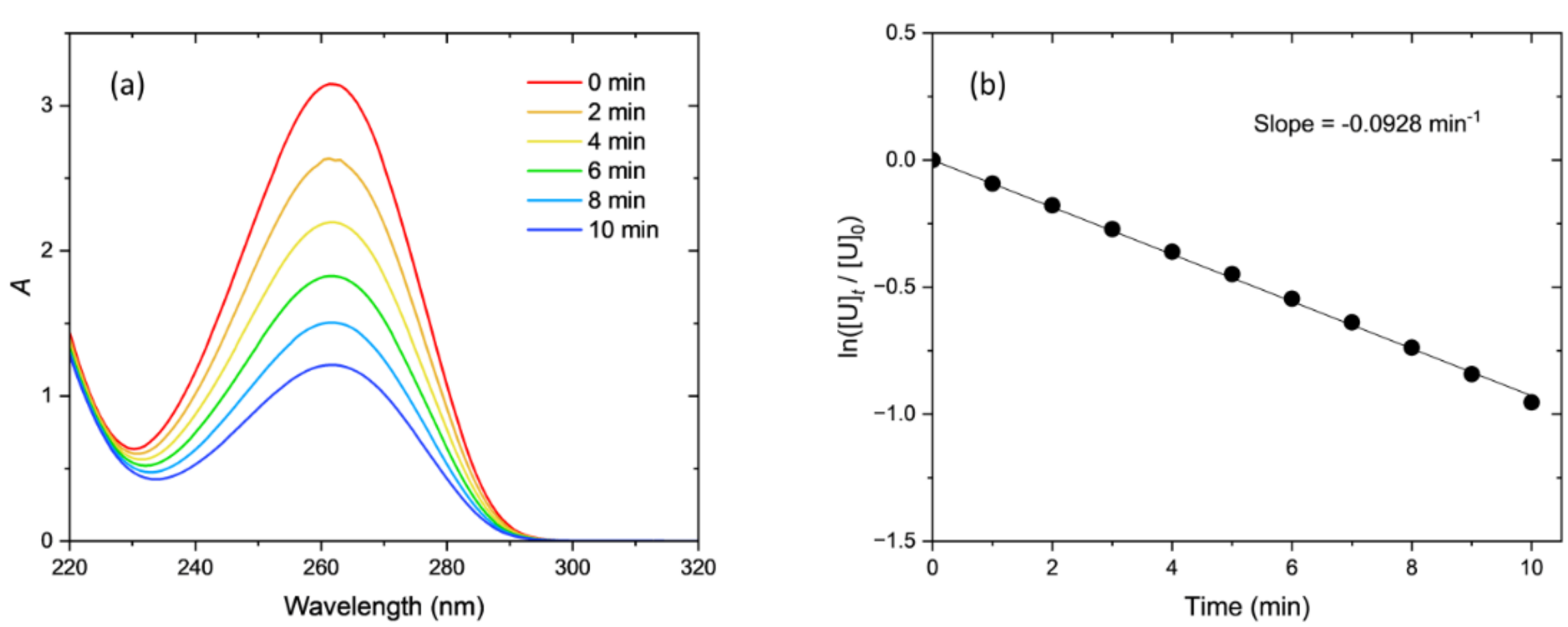

Figure S20. (a) The absorption spectra of uridine in water after irradiating for the indicated times in the Hg lamp photoreactor. (b) Plot of ln([Uridine]$_t$ / [Uridine]$_0$) vs. time (markers) with the best-fit line. The slope of the line is the first-order rate constant in min^{-1}.

11) Additional data fitting

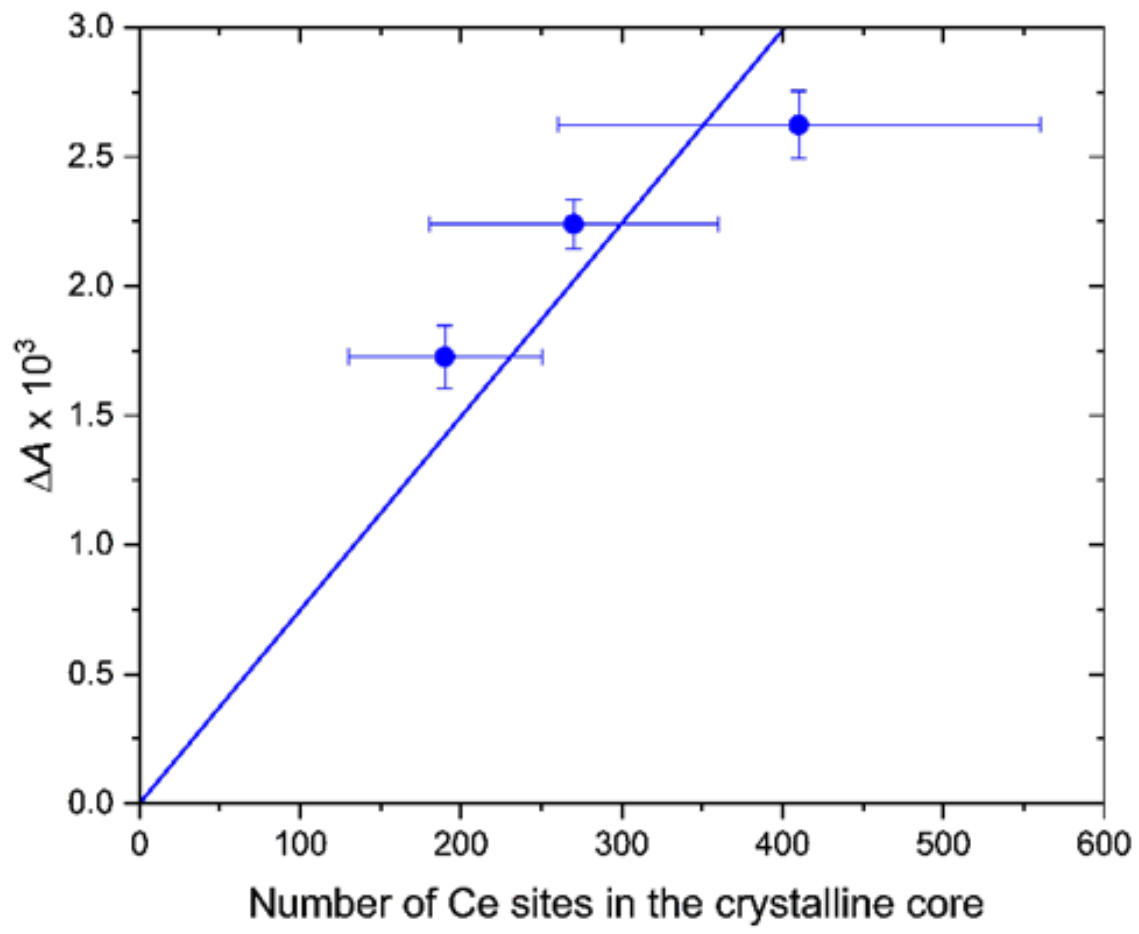

Figure S21. The initial transient amplitude of the UV band plotted against the estimated number of Ce sites in the crystalline core (Table S7) for undoped nanoceria, La-10% nanoceria, and La-20% nanoceria (corresponding to highest, middle, and lowest points accordingly). The y-intercept of the linear fit is fixed at zero. Error bars are 2σ.

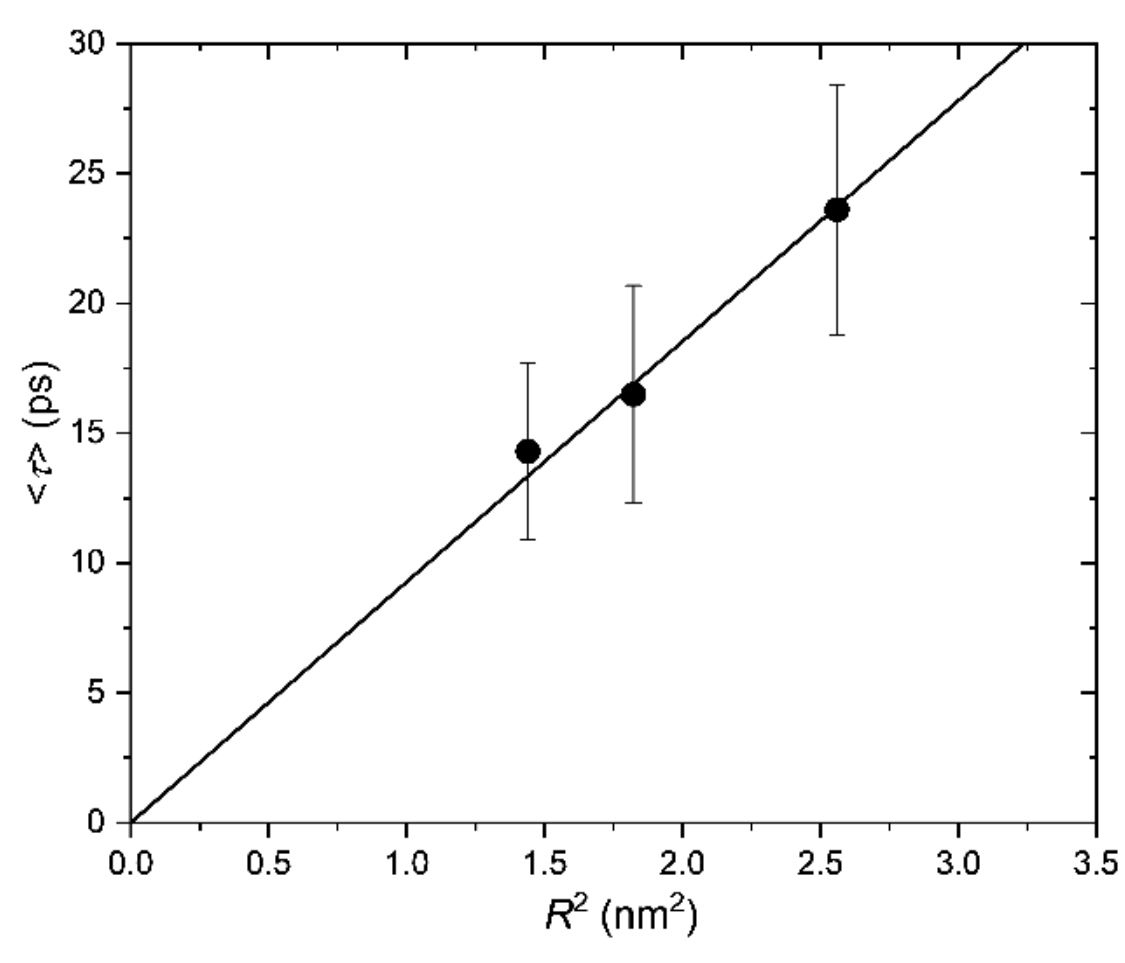

Figure S22. Average transit time for ESPs to reach the nanoparticle surface vs. the nanoparticle radius squared for undoped nanoceria, La-10% nanoceria, and La-20% nanoceria (corresponding to highest, middle, and lowest points accordingly). The y-intercept of the linear fit is fixed at zero. The error bars shown are 2σ.

Eq S15 (see Section S7 in ref 6) shows the relationship between the mean first passage time to the surface in a sphere and the radius of the sphere

$$\langle \tau \rangle = \frac{R^2}{15D} \quad \text{(S15)}$$

The best-fit line passing through the origin has a slope of 9.3 ± 0.4 ps nm^{-2} (2σ). Using eq S15, the slope predicts a diffusion coefficient of $D = (0.7 \pm 0.3) \times 10^{-4}$ cm^2 s^{-1}.

REFERENCES

(1) Asati, A.; Santra, S.; Kaittanis, C.; Nath, S.; Perez, J. M. Oxidase-Like Activity of Polymer-Coated Cerium Oxide Nanoparticles. *Angew. Chem., Int. Ed.* **2009**, *48*, 2308–2312.

(2) Pettinger, N. W.; Empey, J. M.; Fröbel, S.; Kohler, B. Photoreductive Dissolution of Cerium Oxide Nanoparticles and Their Size-Dependent Absorption Properties. *Phys. Chem. Chem. Phys.* **2020**, *22*, 5756–5764.

(3) Ghosh, S.; Pham, B.; Madhu, M.; Kohler, B. Interband- and Intraband-Induced Hot Electron Transfer in Plasmonic Gold–Cerium Oxide Core–Shell Nanoparticles. *J. Phys. Chem. C* **2023**, *127*, 21593–21602.

(4) Schindelin, J.; Arganda-Carreras, I.; Frise, E.; Kaynig, V.; Longair, M.; Pietzsch, T.; Preibisch, S.; Rueden, C.; Saalfeld, S.; Schmid, B.; Tinevez, J.-Y.; White, D. J.; Hartenstein, V.; Eliceiri, K.; Tomancak, P.; Cardona, A. Fiji: An Open-Source Platform for Biological-Image Analysis. *Nat. Methods* **2012**, *9*, 676–682.

(5) Khorsand Zak, A.; Abd. Majid, W. H.; Abrishami, M. E.; Yousefi, R. X-Ray Analysis of ZnO Nanoparticles by Williamson–Hall and Size–Strain Plot Methods. *Solid State Sci.* **2011**, *13*, 251–256.

(6) Pham, B.; Pettinger, N. W.; Kohler, B. Ultrafast Dynamics of Photogenerated Carriers in Cerium Oxide Nanoparticles. *J. Phys. Chem. C* **2026**, *130*, 4109–4123.

(7) Khosrofian, J. M.; Garetz, B. A. Measurement of a Gaussian Laser Beam Diameter through the Direct Inversion of Knife-Edge Data. *Appl. Opt., AO* **1983**, *22*, 3406–3410.

(8) Prieur, D.; Bonani, W.; Popa, K.; Walter, O.; Kriegsman, K. W.; Engelhard, M. H.; Guo, X.; Eloirdi, R.; Gouder, T.; Beck, A.; Vitova, T.; Scheinost, A. C.; Kvashnina, K.; Martin, P. Size Dependence of Lattice Parameter and Electronic Structure in CeO_2 Nanoparticles. *Inorg. Chem.* **2020**, *59*, 5760–5767.

(9) Lindsey, C. P.; Patterson, G. D. Detailed Comparison of the Williams–Watts and Cole–Davidson Functions. *J. Chem. Phys.* **1980**, *73*, 3348–3357.

(10) Witt, C.; Schötz, K.; Köhler, A.; Panzer, F. Understanding Method-Dependent Differences in Urbach Energies in Halide Perovskites. *J. Phys. Chem. C* **2024**, *128*, 6336–6345.

(11) Zatsepin, A. F.; Kuznetsova, Yu. A.; Sokolov, V. I. UV Absorption and Effects of Local Atomic Disordering in the Nickel Oxide Nanoparticles. *J. Lumin.* **2017**, *183*, 135–142.

(12) Pelli Cresi, J. S.; Di Mario, L.; Catone, D.; Martelli, F.; Paladini, A.; Turchini, S.; D'Addato, S.; Luches, P.; O'Keeffe, P. Ultrafast Formation of Small Polarons and the Optical Gap in CeO_2. *J. Phys. Chem. Lett.* **2020**, *11*, 5686–5691.

(13) Rabani, J.; Mamane, H.; Pousty, D.; Bolton, J. R. Practical Chemical Actinometry—A Review. *Photochem. Photobiol.* **2021**, *97*, 873–902.
(14) Kuhn, H. J.; Braslavsky, S. E.; Schmidt, R. Chemical actinometry (IUPAC Technical Report). *Pure Appl. Chem.* **2004**, *76*, 2105–2146.
